# Tuning Pore Size in Porous Graphene Membrane for O_2_/N_2_ Separation

**DOI:** 10.1002/adma.202519645

**Published:** 2025-12-28

**Authors:** Kuang‐Jung Hsu, Marina Micari, Yueqing Shen, Shaoxian Li, Shuqing Song, Kumar Varoon Agrawal

**Affiliations:** ^1^ Laboratory of Advanced Separations École Polytechnique Fédérale de Lausanne (EPFL) Sion Switzerland

**Keywords:** gas separation, membranes, O_2_/N_2_ separation, porous graphene

## Abstract

Graphene with angstrom‐scale, zero‐dimensional pores offers a promising platform for gas separations due to its exceptional permeance and potential for molecular sieving. Herein, we demonstrate a dynamic strategy to tune N‐functionalized graphene pores, achieving selective oxygen (O_2_) separation from nitrogen (N_2_), a particularly challenging separation due to their similar kinetic diameters. We exploit the heterogeneity of functional groups at the pore edge to tune the pore limiting diameter (PLD). By facile thermal annealing, we convert primary amine groups at the pore edge to lattice‐incorporated nitrogen. Temperature‐dependent extent of conversion allows to tune the steric hindrance from amine‐CO_2_ complex, and therefore, PLD for O_2_/N_2_ separation in favor of O_2_ permeation. The resulting membranes exhibit attractive O_2_/N_2_ separation performance, with O_2_ permeance near 2500 GPU with O_2_/N_2_ selectivity above 10, significantly outperforming the state‐of‐the‐art membranes. This is attractive for energy‐efficient and modular production of O_2_ from air and can cut down fuel consumption in natural gas‐fired furnaces in the chemical industry by 60%.

## Graphical abstract

Single‐layer graphene membranes featuring N‐functionalized, 0D pores offer a promising platform for O_2_/N_2_ separation. The heterogeneity of functional groups at the pore edge is dynamically tunable, enabling precise tuning of the pore limiting diameter and gas transport. This strategy achieves attractive performance, with O_2_ permeance approaching 2500 GPU and O_2_/N_2_ selectivity exceeding 10, highlighting the potential for energy‐efficient air separation.

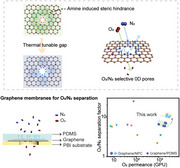

## 1 Introduction

The separation of O_2_ from N_2_ is crucial for the chemical industry [1], energy generation [2], and the on‐site production of medical‐grade O_2_ [3]. To meet energy and sustainability goals, energy‐efficient, decentralized, and modular O_2_ separation processes are needed. Currently, cryogenic distillation is used for large‐scale O_2_ production; however, this process becomes expensive for mid to small‐scale processes [4, 5, 6, 7]. In this respect, processes based on high‐performance membranes, offering high O_2_ permeance as well as a high O_2_/N_2_ selectivity, become attractive.

The performance of the state‐of‐the‐art membranes for O_2_/N_2_ separation has been limited due to challenges in material design for this separation. Commercial membranes based on polymeric films are fundamentally constrained by the trade‐off between permeance and selectivity [8, 9, 10]. Typically, an O_2_/N_2_ selectivity of 3–6 and O_2_ permeance of 10–100 gas permeance units (GPU, 1 GPU = 3.35 **×** 10^−10^ mol m^−2^ s^−1^ Pa^−1^) is obtained [9, 11]. Higher selectivity has been reported using inorganic nanoporous materials, e.g., carbon molecular sieves (CMS) [12, 13] and mixed matrix membranes [14, 15]. However, permeance of these membranes is low due to challenges in engineering an ultrathin selective layer [16]. Facilitated transport membranes have yielded attractive O_2_/N_2_ separation performance [17, 18]. However, they suffer from carrier saturation and stability issues.

Nanoporous atomically thin membranes (NATMs), particularly porous graphene hosting 0D pores [19, 20], are promising for challenging gas separations [21, 22, 23, 24, 25, 26, 27, 28]. Single‐step translocation across the atomically thin pores of graphene enables exceptionally high permeance [29, 30, 31, 32, 33]. When PLD in graphene pores is comparable to molecular diameter, one can achieve high gas permeance as well as high gas pair selectivity [22, 24, 34]. Molecular simulations predict that graphene pores can differentiate between O_2_ (kinetic diameter of 3.46 Å) and N_2_ (kinetic diameter of 3.64 Å) with high selectivity by dynamic electron density gap [4]. Yet, while attractive O_2_ permeance (1000 GPU) has been reported from porous graphene, these membranes have yielded only a modest O_2_/N_2_ selectivity (∼3–4) [24].

The limited O_2_/N_2_ selectivity from porous graphene is due to a lack of methods for fine‐tuning the electron density gap in graphene pores for this challenging separation, given the difference between the kinetic diameters of O_2_ and N_2_ is just 0.18 Å [26, 31, 35]. To achieve a high selectivity, the effective gap in the pore must be small enough to introduce an energy barrier for gas translocation across the pores [20, 36, 37, 38, 39].

**FIGURE 1:**
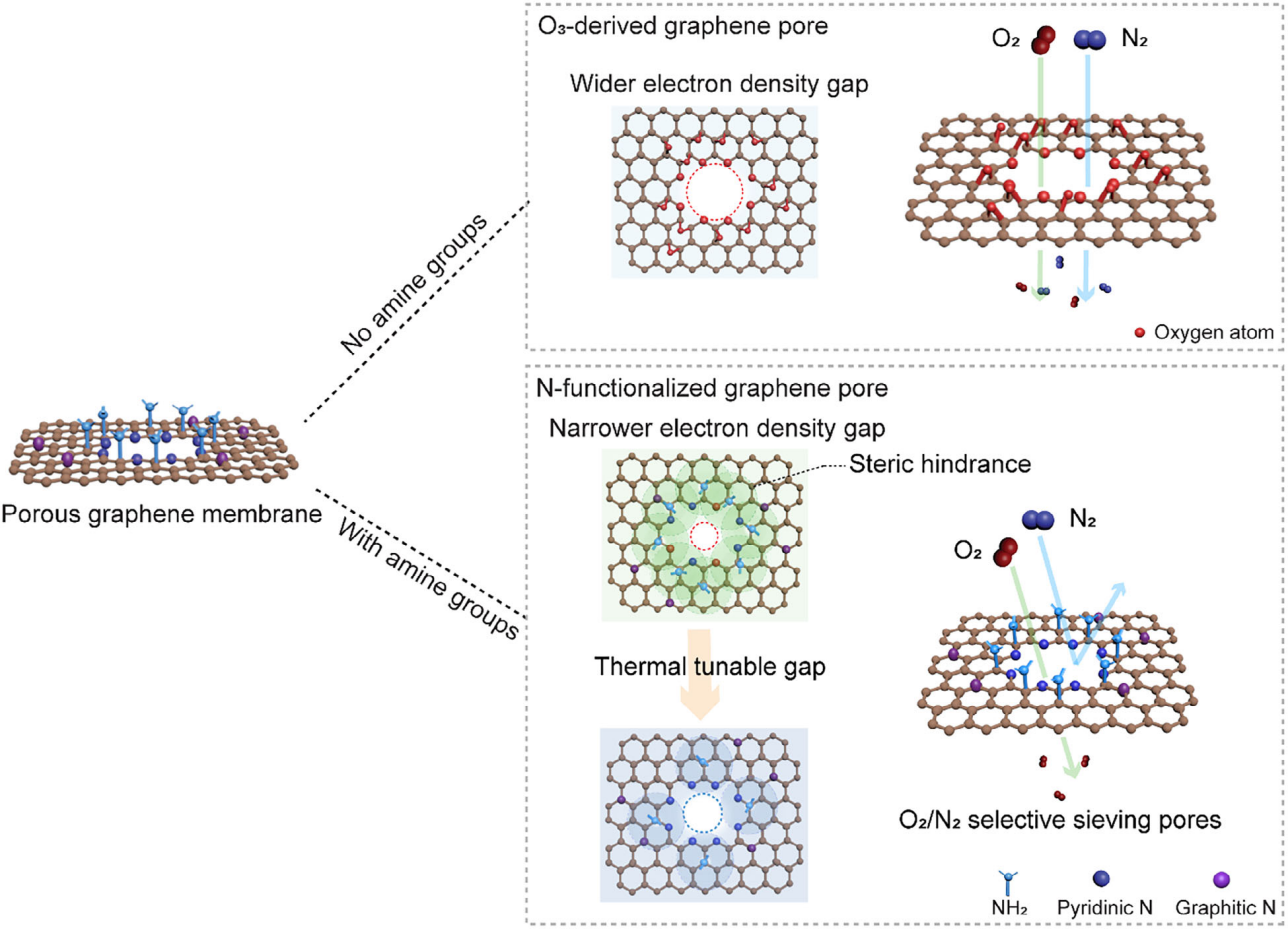
Schematic illustration of graphene with thermally tunable N‐functionalized 0D pore size for selective O_2_/N_2_ separation. O_3_‐derived graphene pores terminated with O atoms have yielded limited O_2_/N_2_ selectivity. When amine groups surrounding the 0D pores introduce steric hindrance to gas transport by narrowing the local electron density gap, thereby enabling O_2_/N_2_ selective transport.

Herein, we demonstrate precise tuning of the effective electron density gap in Å‐scale graphene pores via nitrogen (N) functionalization at the pore edges (Figure 1). By incorporating primary amine (‐NH_2_) adjacent to graphene pores, we leverage their strong CO_2_ chemisorption affinity to form bulky complexes (e.g., ‐NHCOO^−^) [4, 25, 40]. This results in steric hindrance at the pore, effectively narrowing the pore's electron density gap and introducing a sharp molecular cut‐off for O_2_/N_2_ separation [26, 41, 42].

We demonstrate that the electron density gap in the pore can be dynamically tuned through thermal annealing [31, 43], which incorporates primary amines into the graphene lattice as pyridinic N, thereby modifying gas transport. This controllable modulation yields exceptional O_2_/N_2_ separation performance, with an average O_2_ permeance of 2580 ± 399 GPU and selectivity of 10.7 ± 0.6. Notably, centimeter‐scale membranes are demonstrated that maintain stable separation performance over several days.

## 2 Results

### 2.1 O_2_/N_2_ Selective Transport Led by Steric Hindrance From ‐NH_2_

**FIGURE 2:**
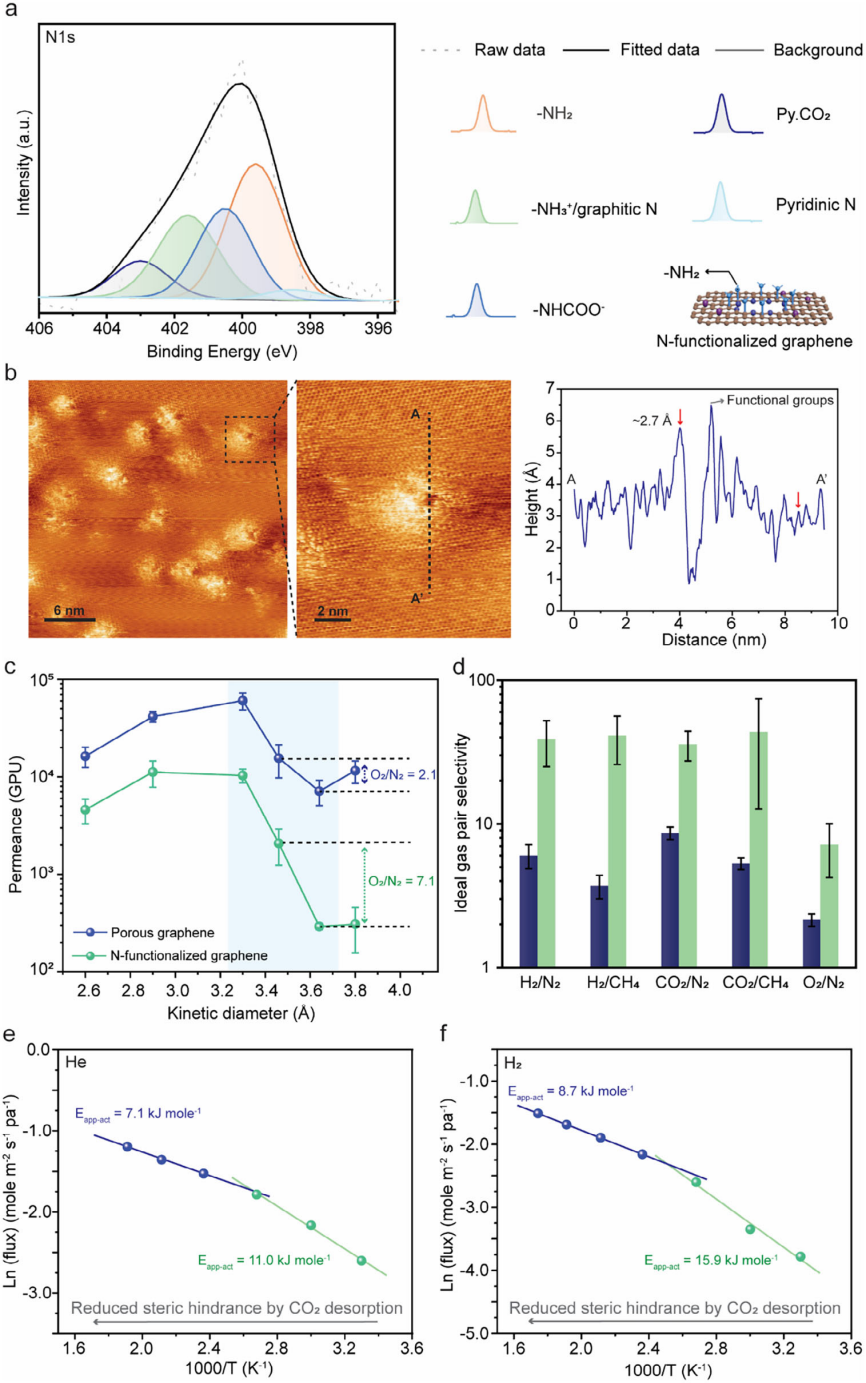
Characterization and gas permeation properties of N‐functionalized graphene. (a) N 1s XPS spectrum of graphene showing –NH_2_ groups, their CO_2_‐bound derivatives, and pyridinic N and CO_2_‐adsorbed pyridinic N (Py.CO_2_). (b) STM image of pores surrounded by N‐functional groups (bright features). Some pores appear blocked, while others display a small gap. The height profile of the line (AA′) in the STM image on the left. (c) Single‐gas permeation measurements and (d) ideal gas pair selectivity of porous graphene derived from O_3_ etching and N‐functionalized graphene/NPC membranes. All gas permeation experiments were conducted at 30°C and 2 bar feed pressure. Error bars represent the standard deviation of permeances and ideal selectivity across three membranes; the center of each bar indicates the average value. (e) Arrhenius plots of single‐gas permeation versus reciprocal temperature for apparent activation energies (*E*_app_) of He and H_2_, based on N‐functionalized graphene/NPC membranes. The decrease in *E*_app_ with increasing temperature is attributed to reduced steric hindrance due to CO_2_ desorption at elevated temperatures. All gas permeation experiments were conducted at 2 bar feed pressure.

Pores in chemical vapor deposition (CVD)‐derived single‐layer graphene were incorporated by controlled ozone (O_3_) oxidation (Figure S1), as described in the literature [44]. This approach results in epoxy clusters with a pore at the center of the cluster hosting ether and semiquinone groups at the pore edge [45, 46]. Incorporation of N‐functional group was carried out by ammonia (NH_3_) treatment at 20°C for 24 h [25]. The semiquinone and epoxy groups react with NH_3_ to form pyridinic N and ‐NH_2_, respectively [25]. The affinity of ‐NH_2_ toward CO_2_ results in derivatives (NHCOO^−^ and ‐NH_3_^+^ groups), as indicated by X‐ray photoelectron spectroscopy (XPS, Figure 2a). The density of ‐NH_2_ and its derivatives is estimated to be 3.0 × 10^14^ cm^−2^, based on N composition (∼9%). These pores were visualized using low‐temperature scanning tunneling microscopy (LTSTM, Figure 2b) of a graphitic sample prepared in the same manner. Bright features surrounding the pores correspond to the functional groups. These functional groups lead to steric hindrance at the pores. Several pores were observed to be blocked (Figure S2), while others exhibited a narrow gap (Figure 2b).

To investigate the effect of steric hindrance from ‐NH_2_ and its derivatives on gas transport, suspended graphene membranes were prepared. Briefly, a thin mechanically‐reinforcing film (MRF) of nanoporous carbon (NPC) was deposited on porous graphene (Figure S3). Subsequently, graphene was transferred from the CVD substrate (Cu) onto a macroporous membrane support [47]. The standalone NPC film yields a large nonselective gas flux (Figure S4) and does not limit the transport from graphene. This allows for exploring the gas transport properties of porous graphene. A single‐component gas permeation test was carried out by pressurizing the feed side of the membrane to 2 bar with the desired gas and sweeping the permeate side with argon (Ar). Before N‐functionalization, porous graphene yielded a high O_2_ permeance (> 15000 GPU) with a small O_2_/N_2_ ideal selectivity (2.1). Following N‐functionalization, there was a drastic improvement in O_2_/N_2_ ideal selectivity (7.1), accompanied with an attractive O_2_ permeance (> 2000 GPU). To further understand the steric hindrance of the pores, single‐component permeation tests with several gases, with increasing kinetic diameter, were conducted at a feed pressure of 2 bar (He, H_2_, CO_2_, O_2_, N_2_, CH_4_). The permeance of all gases was reduced; however, there was a noticeable decrease in the permeance of N_2_ and CH_4_ (Figure 2c), resulting in higher selectivity with respect to these gases (Figure 2d), which indicates that steric hindrance effectively reduced pore size. To further understand steric hindrance and its effect on narrowing the electron density gap, temperature‐dependent single‐gas permeation measurements were carried out in the range of 30°C–300°C. He and H_2_ were selected as probe gases because of their negligible binding energies. To eliminate interference with the evolution of N‐functional groups (discussed later), the membranes were annealed at 300°C before permeation measurement. The permeance of He and H_2_ was temperature‐activated, confirming that transport occurs through narrow pores where gas translocation takes place by overcoming an energy barrier. The temperature‐dependent gas flux, when plotted as per the Arrhenius relationship, displayed two different slopes, with a transition at ∼120°C (Figure 2e,f). The apparent activation energy (*E*_act‐app_) for H_2_ (He) decreased from 15.9 kJ mol^−1^ (11.0 kJ mol^−1^) to 8.7 kJ mol^−1^ (7.1 kJ mol^−1^) at higher temperatures. This indicates the larger electron density gap at elevated temperature. This is attributed to the fact that the population of the ‐NH_2_ derivatives (NHCOO^−^ and ‐NH_3_^+^), formed by CO_2_ chemisorption, decreases at elevated temperatures, which then reduces steric hindrance at the pores (Figure S5).

### 2.2 Dynamic Thermal Tuning Pore Limiting Diameter

**FIGURE 3:**
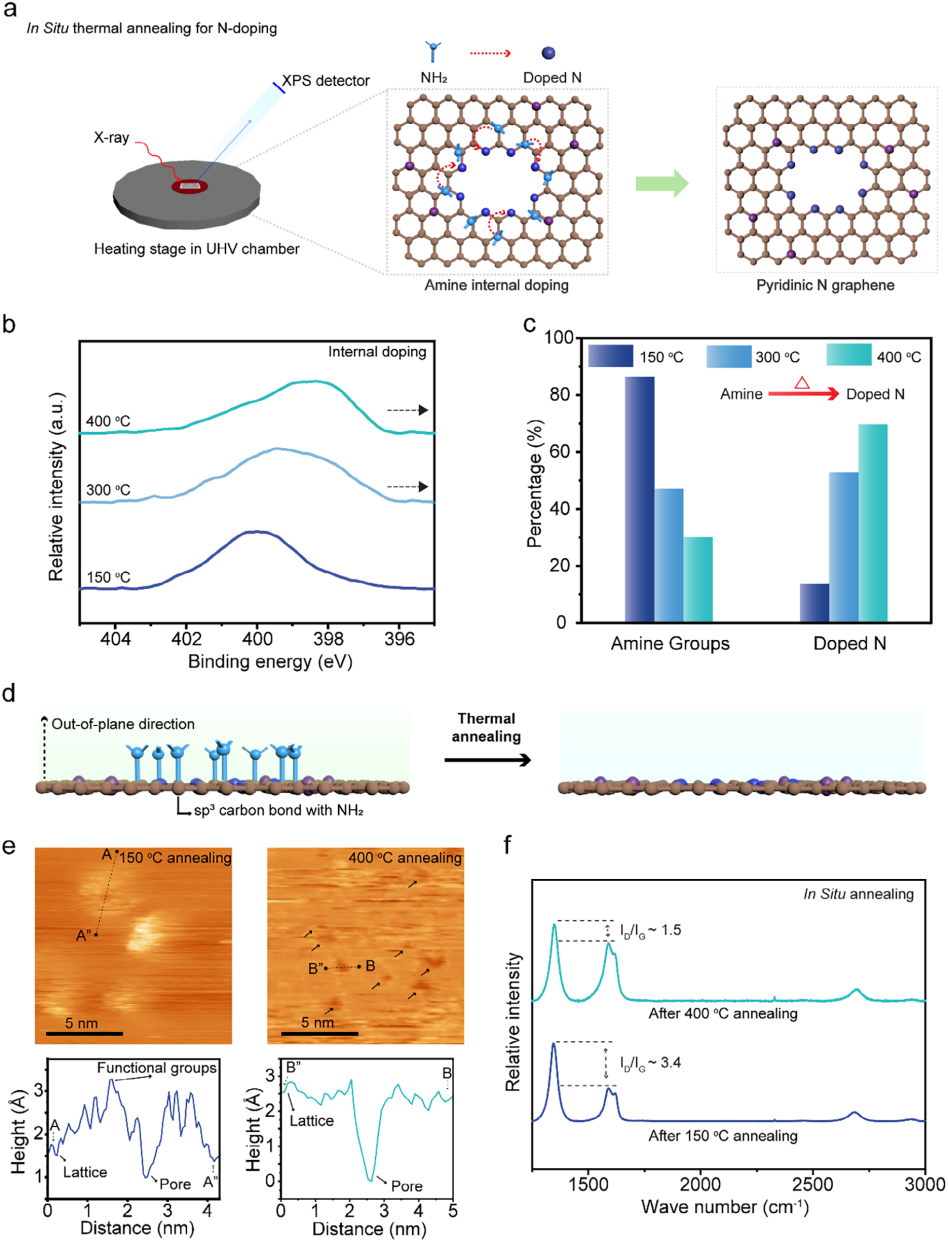
Characterization of thermally tunable N‐functionalized 0D pores. (a) Schematic illustration showing in Situ thermal annealing inside the XPS UHV chamber allows for monitoring the conversion of –NH_2_ groups into pyridinic and graphitic N. (b) N 1s XPS spectra and (c) corresponding quantification of amine and doped nitrogen species, including pyridinic N and graphitic N, after annealing at 150°C, 300°C, and 400°C. (d) Schematic illustration depicting amine groups positioned out‐of‐plane around the 0D pores, while doped N atoms are incorporated into the graphene lattice. The schematic illustration explains that the bright features disappeared after the thermal annealing in the following STM images. (e) STM image and corresponding height profile of N‐functionalized 0D pores following thermal annealing at 150°C and 400°C for 1 h in the UHV chamber, showing that the out‐of‐plane functional groups were converted during the annealing process. The arrows point at the 0D pores on the graphene lattice. (f) Raman spectra of N‐functionalized graphene after thermal annealing at 150°C and 400°C for 1 h.

We observed that ‐NH_2_ functional groups, which surround graphene pores, were transformed into lattice N during thermal annealing of the sample (Figure 3a). This is attributed to a higher thermodynamic stability of pyridinic and graphitic N [43, 48, 49]. This transformation is expected to increase the effective electron density gap (PLD) due to the smaller steric profile of lattice N, especially because they do not chemisorb CO_2_ [25]. To investigate this evolution, we conducted in situ thermal annealing inside the XPS chamber (Figure 3b). Annealing was carried out for 1 h at 150°C, 300°C, and 400°C to understand the role of steric hindrance. XPS N1s spectra revealed progressive peak shifts with increasing temperature, while the overall N content remained constant (∼9%) (Figure S6). Deconvolution of the N 1s peaks revealed that amine groups were gradually converted into pyridinic and graphitic N species (Figure 3c; Figure S7), confirming chemical transformation rather than loss of N.

LTSTM was used to visualize changes in the pore morphology during thermal annealing. At 150°C, STM images revealed bright, clustered features, consistent with the presence of ‐NH_2_ and derivative functional groups protruding out of the graphene plane (Figure 3d,e) [50, 51, 52, 53]. After annealing at 400°C, these clusters diminished in size. This change reflects the conversion of out‐of‐plane amine derivatives to in‐plane lattice nitrogen, which reduces steric hindrance within the pore [54]. Therefore, the transformation of ‐NH_2_ groups at the pore edges into lattice nitrogen increases the electron density gap.

To further probe structural changes during thermal annealing, Raman spectroscopy of porous graphene was performed (Figure S8). For this, porous graphene was transferred to Si/SiO_2_. Upon thermal annealing to 400°C, the *I_D_/I_G_* ratio decreased significantly (from ∼3.4 to ∼1.5, Figure 3f), consistent with the transformation of sp^3^‐hybridized carbon bonded to amines into sp^2^‐hybridized, N‐doped graphene. Since dopant‐related defects are Raman‐inactive (silent defects) [55, 56, 57], the reduced *D* peak intensity reflects this conversion. Additionally, shifts in the positions of the *G* peak (ω_*G*_) and *2D* peak (ω_2*D*_) indicate a change in doping state. Pyridinic N induces *p*‐type doping, while graphitic N favors *n*‐type behavior [58, 59, 60]. Raman data after 400°C annealing showed a shift toward *p*‐type doping (Figure S9), indicating the prevalent formation of pyridinic N, which is confirmed by the XPS data.

**FIGURE 4:**
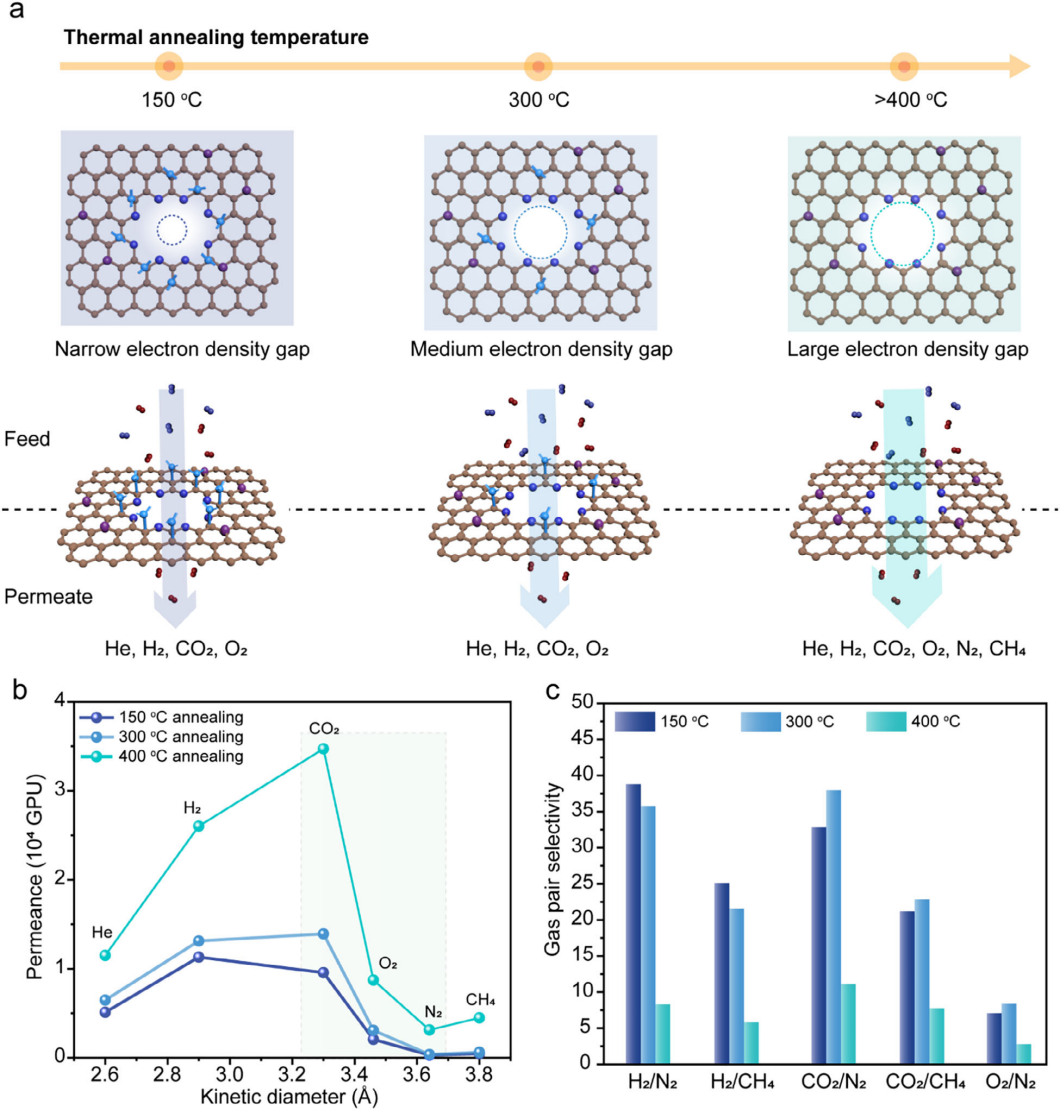
Gas transport properties of thermally tunable N‐functionalized graphene 0D pores. (a) Schematic illustration showing how thermal annealing at 150°C, 300°C, and 400°C tunes the electron density gap size around the pores. (b) Single‐gas permeation measurements and (c) gas pair selectivity of N‐functionalized graphene/NPC membrane after thermal annealing at different temperatures. All gas permeation experiments were conducted at 30°C and 2 bar feed pressure.

The amine conversion during the thermal annealing leads to a gradual enlargement of the electron density gap inside the 0D graphene pores (Figure 4a). To evaluate the evolution of the electron density gap upon annealing, we carried out single‐gas permeation at 30°C and 2 bar feed pressure, using porous graphene annealed at 150°C, 300°C, and 400°C for 1 h. The results showed a significant increase in the permeances of He, H_2_, CO_2_, and O_2_ after annealing at 300°C compared to those annealed at 150°C, whereas N_2_ and CH_4_ permeance exhibited only a slight increase (Figure 4b). This indicates a sharpened molecular cut‐off, particularly between CO_2_ (kinetic diameter of 3.3 Å) and N_2_ (3.64 Å). Specifically, O_2_ permeance increased from 2070 to 3075 GPU, while O_2_/N_2_ selectivity improved from 7.1 to 8.4 (Figure 4c). A similar trend was also observed for porous graphene prepared under milder oxidation conditions (referred to as the “mild oxidation” condition), which hosts lower porosity (Figures S10 and S11 and Note S3). These results demonstrate that thermal annealing enables fine‐tuning of pore properties by changing edge chemistry, offering a scalable and straightforward route to enhance gas separation performance.

For samples annealed to 400°C, gas permeance increased significantly for all tested gas components (Figure 4b), indicating the formation of a higher density of permeable pores. This is attributed to the continued conversion of amine groups into nitrogen dopants, which results in larger effective pore openings and reduced steric hindrance. Notably, CO_2_ permeance exceeded H_2_ permeance after annealing at 400°C (Figure 4b; Figure S12). This is primarily due to the enlarged electron density gap, which lowers the energy barrier for CO_2_ translocation, while the higher affinity of CO_2_ to the pyridinic‐N‐terminated pore edges promotes its permeation [25]. The *E*_act‐app_ derived from temperature‐dependent permeation measurements, can be expressed as the sum of the activation energy (*E_act_*) and the adsorption enthalpy (*H_ad_*).

(1) $$E_{app-act}=E_{act}+H_{ad}$$

By substituting *H_ad_* (‐25.2 kJ mole^−1^) for CO_2_ on pyridinic‐N substituted graphene pores [25], *E_act_* for CO_2_ was calculated. When the sample was annealed at 150°C, an *E_act_* of 27.2 kJ mol^−1^ was obtained. This decreased to 23.5 kJ mol^−1^ after annealing at 300°C (Table S1). This trend further confirms that thermal annealing reduces the energy barrier for gas transport, consistent with the formation of larger, less obstructed pores.

### 2.3 Transport Modeling for Understanding the Effective Pore Structure

**FIGURE 5:**
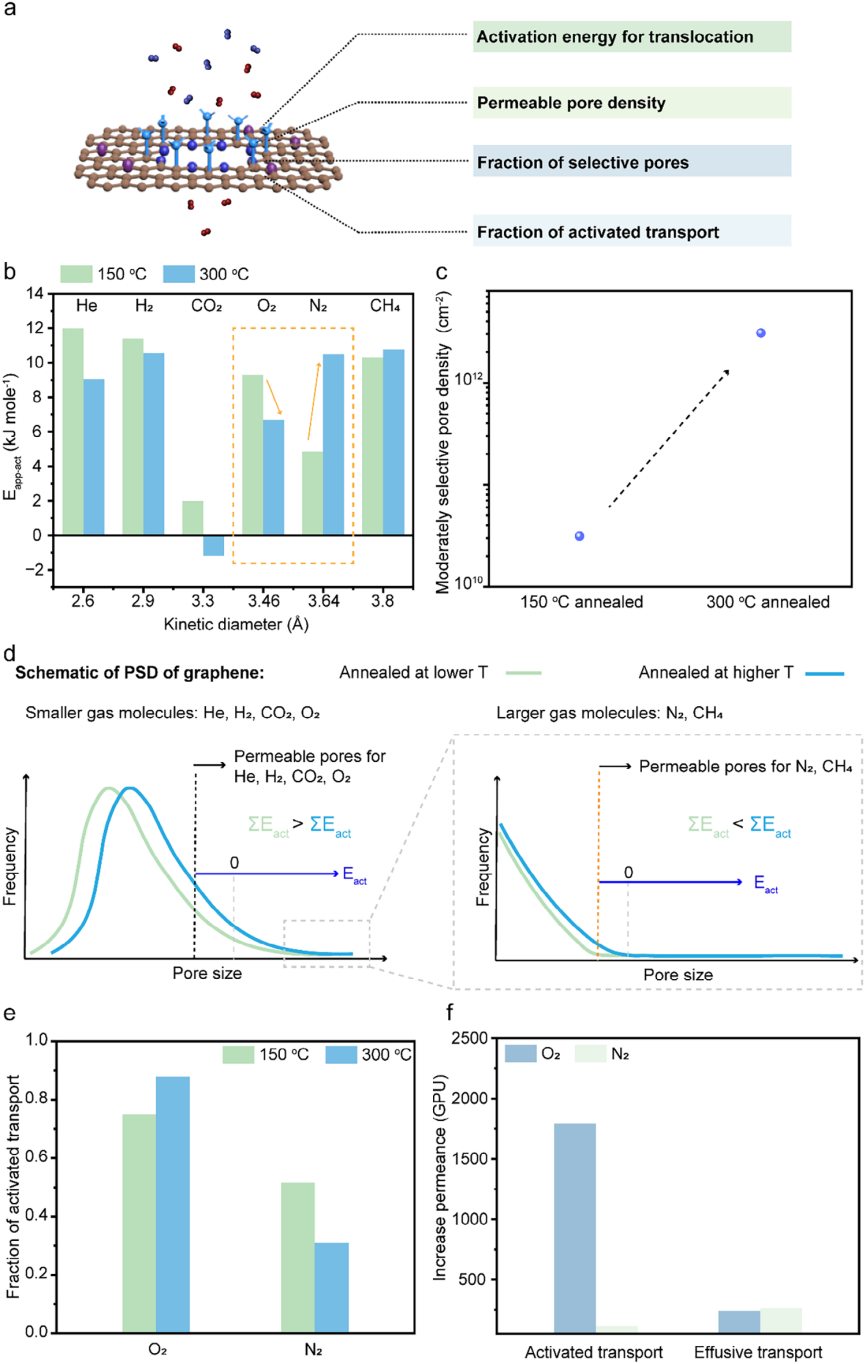
Gas transport properties and gas transport modeling of graphene 0D pores after thermal annealing. (a) Schematic illustration highlights key factors that govern gas transport across graphene 0D pores, including activation energy for translocation, permeable pore density, and the fraction of activated versus effusive transport. (b) Apparent activation energies for various gases extracted from permeation data using the Arrhenius relationship. The gas permeation measurement was conducted after thermal annealing at 150°C and 300°C. (c) Model‐derived moderately selective pore density in graphene membranes annealed at different temperatures. (d) Schematic illustration showing how the pore size distribution (PSD) of graphene evolves after thermal annealing at lower and higher annealing temperatures, resulting in different trends in activation energy for smaller gas molecules (He, H_2_, CO_2_, O_2_) and larger gas molecules (N_2_ and CH_4_). (e) Model‐derived fractions of activated transport after thermal annealing at 150°C and 300°C. (f) The model‐derived O_2_ and N_2_ permeance differences, attributed to activated and effusive transport, were compared for graphene membranes annealed at 150°C and 300°C. The porous graphene membrane was prepared by the low‐oxidation O_3_ etching condition before N‐functionalization.

To gain deeper insight into gas transport mechanisms and effective pore structure in graphene membranes following thermal annealing, we developed a mathematical model to describe the permeation behavior (Figure 5). In this model, total gas permeance is considered to arise from two parallel pathways; selective transport through Å‐scale pores where translocation is the rate‐limiting step [35, 61], and effusive transport through non‐selective pores, governed primarily by the resistance from the membrane support [62]. Modeling was conducted on graphene membranes with low pore density prepared using mild oxidation. Graphene with low pore density was chosen because high pore density often results in pore–pore coalescence during thermal annealing, as O functional groups on the graphene lattice are gasified [63]. Lower pore density minimizes such coalescence, thereby allowing the model to more accurately estimate the contribution of nonselective pores due to reduced steric hindrance.

For selective Å‐scale pores, permeance for a given pore (*P_sel_*, unit of mol s^−1^ Pa^−1^) is determined by *E*_act‐app_ required for gas translocation [26, 27]. Therefore, the selective Å‐scale pores can be categorized into two pore classes (Note S2 and Figure S15). (1) small, highly selective pores where transport requires high *E*_act‐app_ leading to lower permeance (*P*_*sel*, *s*_). (2) larger, moderately selective pores with faster transport across pores requiring a lower *E*_act‐app_ for translocation, leading to higher permeance (*P*_*sel*, *m*_). The total gas permeance from selective pores (*P*_*T*, *sel*_) can be obtained by summing transport from all selective pores, using the number density of pores (ρ_*sel*, *s*_ and ρ_*sel*, *m*_, unit of cm^−2^, Equation (2)).

(2) $$P_{T,\;sel}=\;\rho_{sel,\;s}P_{sel,\;s}+\;\rho_{sel,\;m}P_{sel,\;m}$$

On the other hand, the gas transport resistance across the larger, non‐selective area in the membrane is determined by the resistance of the support layer. Therefore, the gas permeance from the non‐selective area (*P*_*T*, *nonsel*_) can be approximated as the permeance of the support layer (*P_support_*). Denoting the nonselective fractional area by θ_*nonsel*_, one gets the effective permeance of nonselective part of the membrane as

(3) $$P_{T,nonsel\;}=\theta_{nonsel}\;P_{support}$$

where θ_*nonsel*_ varies between 0 and 1, with 0 referring to a perfectly selective membrane and 1 referring to a perfectly nonselective membrane. The total permeance is then

(4) $$P_{T}=P_{T,\;sel}+P_{T,nonsel\;}$$

(5) $$P_{T}=\rho_{sel,\;s}P_{sel,\;s}+\rho_{sel,\;m}P_{sel,\;m}+\theta_{nonsel}\;P_{support}$$

Recently, molecular dynamics simulation and transition‐state theory have been successfully used to predict the permeability coefficient for highly selective and moderately selective functionalized pores (Table S2) [64]. Given that the total permeance contribution from small, highly‐selective pores is marginal, we first assumed the total permeance is mainly contributed from moderately selective and non‐selective pores. Therefore, Equation (5) can be revised into Equation (6).

(6) $$P_{T}=\rho_{sel,\;m}P_{sel,\;m}+\theta_{nonsel}\;P_{support}$$

By substituting the O_2_ and N_2_ permeability coefficients into Equation (6) and regressing against the measured permeance values, we determined ρ_*sel*, *m*_ and θ_*nonsel*_ for membranes annealed at 150°C and 300°C. We can calculate the ρ_*sel*, *s*_ by inserting the obtained ρ_*sel*, *m*_ and θ_*nonsel*_ into Equation (5). Additionally, using temperature‐dependent permeation data, we extracted values of *E*_*app* − *act*_ for each gas and estimated *D_p_* ​ from gas‐pair selectivity [26, 63].

Annealing at 300°C led to a decrease in *E*_*app* − *act*_ for He, H_2_, CO_2_, and O_2_, while *E*_*app* − *act*_ for N_2_ and CH_4_ showed a slight increase (Figure 5b). This suggests that the increased density of the moderate selective pore sizes and the effective pore size distribution shifted to favor smaller gas molecules while still restricting larger ones (Figure 5d). Notably, the calculated *E*_*app* − *act*_ for CO_2_ became negative after annealing at 300°C, indicating a stronger contribution of the negative *H_ad_* with reducing *E_act_*, consistent with the observed CO_2_/H_2_ selectivity reversal (Figure S12).

Due to the lower activation energy for translocation across moderately selective pores, the selective transport is mainly governed by the transport through moderately selective pores (Figure S16), we probed its pore density. The model further reveals that annealing at 300°C increased the pore density relative to 150 °C (Figure 5c), accompanying an increase in O_2_ permeance through moderate selective pores and *D_p_* (Figures S16b and S17). This shift was accompanied by an increase in the fraction of nonselective pores from ∼10 to ∼330 ppm (Figure S17). Despite this, O_2_ permeance remained predominantly governed by activated transport (Figure 5e), confirming that most pores remained selective to smaller gas molecules against N_2_ and CH_4_.

At 150°C, the membrane prepared by mild oxidation exhibited low gas permeance (e.g., 20 GPU for O_2_ and 16 GPU for N_2_) (Figure S18), reflecting that the lognormal pore size distribution had many non‐permeable pores [44]. Following annealing at 300°C, the permeance of O_2_ increased significantly to 2060 GPU, whereas that for N_2_ increased by a relatively lower amount (Figure 5f; Figure S19). This indicates that the population of O_2_/N_2_ selective pores increased substantially more than that compared to the non‐selective pores (Figure 5c; Figure S19), consistent with the decrease (increase) of *E*_*app* − *act*_ for O_2_ (N_2_). As a result, O_2_/N_2_ separation performance improved notably (see discussion in the next section).

### 2.4 O_2_/N_2_ Separation Performance

**FIGURE 6:**
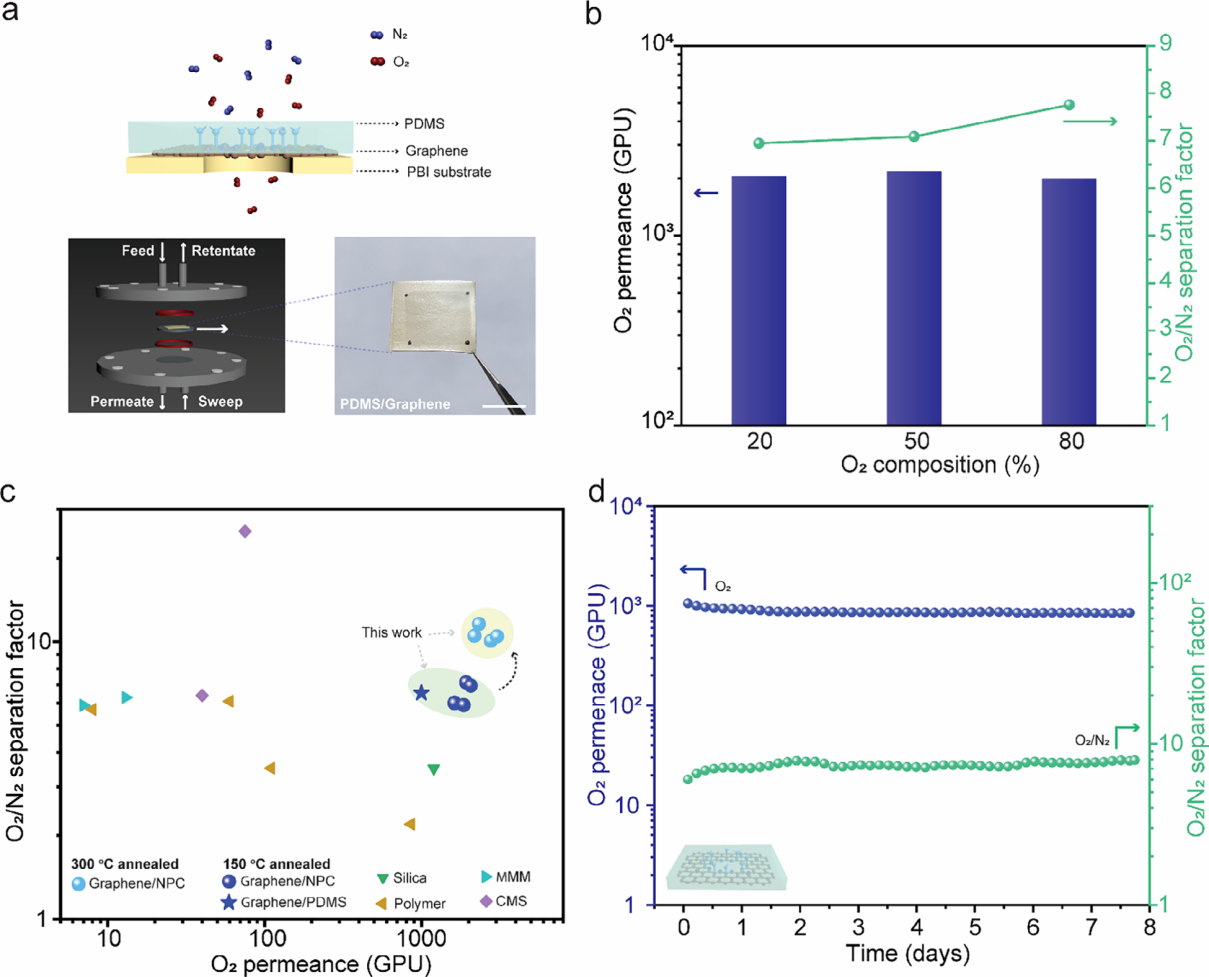
Thermally tunable N‐functionalized graphene 0D pores enable enhanced O_2_/N_2_ separation performance. (a) Schematic illustration of centimeter‐scale graphene/PDMS membranes used for O_2_/N_2_ separation. The scale bar is 1 cm. (b) O_2_/N_2_ separation performance of N‐functionalized graphene membranes under varying O_2_ compositions (20%, 50%, and 80%). (c) Comparison of O_2_/N_2_ mixture separation performance with state‐of‐the‐art membranes. The performance of N‐functionalized graphene membranes is highlighted in the green‐ and yellow‐shaded regions. The graphene membranes supported by NPC (PDMS) were presented by the circular (star) shape points. (d) Stability test of N‐functionalized graphene membranes using a mixed feed (20 vol.% O_2_ in N_2_), demonstrating stable separation performance over time. All gas permeation experiments were performed at 30°C and 2 bar feed pressure.

Tuning the electron density gap in Å‐scale graphene pores enables molecular cut‐off for O_2_/N_2_ separation. We systematically studied this separation using two types of mechanically reinforcing support films: NPC as described earlier, and 250‐nm‐thick polydimethylsiloxane (PDMS, Figures S20 and S21). The PDMS support, known for its flexibility and scalability, allowed the fabrication of centimeter‐scale membranes (Figure 6a; Figure S22). The high O_2_ permeance of both support layers (above 10000 GPU) does not provide significant resistance to the gas transport, the gas permeation was governed primarily by the graphene selective layer (Figure S23).

To assess the sharpness of the molecular cut‐off, we evaluated the separation performance under varying O_2_ composition (20%, 50%, and 80%) in an O_2_/N_2_ mixture. The graphene membrane showed consistent separation performance across the tested partial pressures (Figure 6b; Figure S24), confirming that transport is dominated by molecular sieving. The membranes achieved an average O_2_ permeance of 2580 ± 399 GPU and an O_2_/N_2_ separation factor of 10.7 ± 0.6 (Figure 6c), significantly surpassing the performance of state‐of‐the‐art materials, including CMS [12, 13, 65], polymer thin‐film composites [11, 66, 67, 68, 69], mixed‐matrix membranes [70], and stacked nanosheets [71]. Importantly, the graphene membrane also demonstrated stable separation performance over multiple days (Figure 6d), highlighting its robustness and scalability for practical air separation applications.

### 2.5 Technoeconomic Analysis for the Production of O_2_‐Enriched Air

**FIGURE 7:**
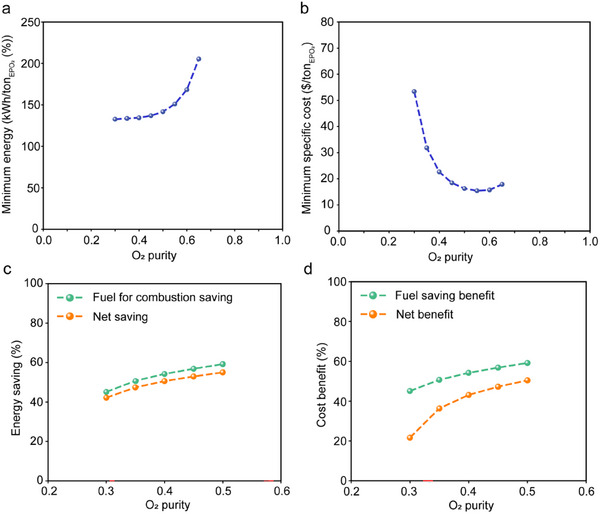
Techno‐economic analysis on the production of oxygen‐enriched air for natural gas combustion. (a,b). Energy consumption and specific costs of a single membrane stage for variable O_2_ purity targets and optimal recovery. Fixed production of 10 ton_EPO2_/day, *P*_feed_ of 1 bar, variable *P*_perm_ and membrane area. (c,d). Net energy savings and cost benefit when oxygen‐enriched air at variable purity is used for natural gas consumption.

Guidelines based on techno‐economic analyses, for developing the membrane process for O_2_‐enriched air, indicate that O_2_/N_2_ selectivity of 10 is optimal for producing mid‐purity O_2_‐enriched air [7], attractive to improve the efficiency of natural gas combustion [2]. Following this, we simulated a single membrane stage for O_2_ enrichment between 30% and 65%, based on the performance of the best membrane in this work, i.e., O_2_ permeance of 2580 GPU and O_2_/N_2_ selectivity of 10.7. The techno‐economic model is presented in Note S4. Membrane area and permeate pressure are considered as decision variables, with feed pressure fixed at 1 bar. O_2_ recovery of 20% was found to be optimal for minimizing energy consumption across all enrichment concentrations investigated (Figure S25). The minimum energy is almost constant for purity below 50%, and then increases steeply when purity increases (Figure 7a). This is because the vacuum level needed on the permeate side increases to achieve higher purities. At the same time, the higher driving force given by the lower pressures yields the reduction of membrane area. For these reasons, the specific costs first decrease and then increase, reaching a minimum in correspondence to purity of 50% (Figure 7b). Based on these results, we calculated the energy and cost benefits of using the single membrane stage to enrich air for fuel combustion. Consistent with the literature, we considered a furnace operating at 1649°C, for which the percentage of natural gas savings is reported as a function of oxygen concentration [2]. Details on combustion efficiency and cost assumptions are reported in Note S5.

The increase in oxygen concentration up to 50% results in natural gas (fuel) savings up to around 60%. To calculate the net energy and cost benefits, we considered the energy required for oxygen enrichment. The energy requirement increases with a higher degree of enrichment. However, since the energy requirement does not change significantly for enrichment below 50% (Figure 7a), the trend of net energy benefit follows that of the fuel saving (Figure 7c), and the highest energy saving corresponds to the highest purity. Similarly, the costs benefit is maximum for 50% purity, which corresponds to the lowest enrichment costs (Figure 7d). Since the new generations of burners support combustion in the presence of enriched air with O_2_ concentration of 45%–50% [72], the proposed membranes are attractive as they can ensure energy and cost reduction up to 55% and 50%, respectively, thanks to the high performances in comparison with state‐of‐the‐art polymeric membranes [2].

## 3 Conclusion

We demonstrate a dynamic and facile strategy to precisely tune Å‐scale pores in graphene for high‐performance O_2_/N_2_ separation, showing the promise of 0D Å‐scale pores in graphene for challenging molecular separations. By introducing sterically active amine groups and subsequently converting them into lattice nitrogen through controlled thermal annealing, we achieved fine modulation of the local electron density gap at the pore edge. This enabled us to find an optimal treatment for a sharp molecular cut‐off between O_2_ and N_2_, yielding membranes with exceptional performance, outperforming the current state‐of‐the‐art, and advancing the prospect of graphene membranes, where only a small O_2_/N_2_ selectivity could be achieved in the past. The simple thermal annealing strategy to adjust steric hindrance at the pore offers a new pathway to rationally control gas transport through atomically thin membranes. Our approach provides a promising route for modular, energy‐efficient air separation technologies.

## 4 Experimental Section

### 4.1 Graphene Synthesis

Single‐layer graphene was synthesized via chemical vapor deposition (CVD) on annealed Cu foil (Strem Chemicals Inc., 99.9% purity, 50 µm thickness). Prior to graphene synthesis, the Cu foil underwent an annealing process in the CVD furnace. First, the Cu foils were rinsed with acetone and isopropanol before being placed into the furnace. The furnace was then filled with 700 Torr of CO_2_ and heated to 1000°C to remove organic contaminants. Following this thermal cleaning step, a 9 vol.% H_2_ in an H_2_/Ar gas mixture was introduced into the furnace at approximately 700 Torr after evacuating CO_2_. The annealing temperature was set to 1075°C and maintained for 4 h. After annealing, the furnace was cooled to 1000°C.

Once the furnace reached 1000°C, a gas mixture containing 25% H_2_ in 32 sccm of H_2_/CH_4_ mixture was introduced. The synthesis process was conducted at 460 mTorr for 30 min. After synthesis, the CH_4_ flow was switched off, and the system was allowed to cool naturally to room‐temperature. The synthesized graphene/Cu was then taken out of the furnace chamber and stored in a vacuum desiccator until further use.

### 4.2 Ozone Etching for Incorporating Å‐Scale Pores

Å‐scale pores on graphene were introduced by O_3_ oxidation reaction at 250°C for a total duration of 3 s [44, 73]. To further increase the density of the permeable pores on graphene samples, the graphene was exposed to O_3_ gas at 20°C for 30 and 60 min [74, 75]. Two different conditions of the graphene samples were prepared, the graphene samples prepared by mild oxidation time (30 mins of O_3_ expansion at 20°C) host lower porosity, and those prepared by high oxidation time (60 mins of O_3_ expansion at 20°C) have higher porosity. The ozone expansion leads to different pore size distribution and molecular cut‐off.

### 4.3 Introducing N‐Functional Groups on 0D Pores

The oxidized graphene was treated with NH_3_ vapor to incorporate N‐functional groups on the graphene lattice, as we prepared previously [25]. The oxidized graphene was transferred to a porous support or silicon wafer before the NH_3_ treatment. Prior to the transfer, the graphene film on Cu was coated with different support layers for different purposes. For probing the gas permeation properties, the graphene film was spin‐coated with NPC and PDMS layers. A solution of 0.2 g of turanose (Sigma–Aldrich) and 0.1 g of polystyrene‐co‐poly(4‐vinyl pyridine) (Polymer Source Inc.) in 2 g of dimethylformamide (DMF, Sigma–Aldrich) was spin‐coated on top of graphene on Cu (1000 r.p.m. for 60 s) for preparing NPC‐reinforced graphene [47]. Following that, the coated film was pyrolyzed at 500°C in the H_2_/Ar environment. The PDMS solution was prepared by dissolving 10 wt.% PDMS (Sylgard 184, containing the monomer and cross‐linking agent) in heptane, with a monomer‐to‐cross‐linking agent ratio of 11:1. The mixture was stirred at 70°C for 4 h and then diluted to 4 wt.%. The resulting solution was spin‐coated onto the porous graphene at 1000 rpm for 1 min, followed by drying at 60°C for 24 h. For the characterization, poly(methyl methacrylate) (PMMA) solution was used as a transfer support film. The solution was spin‐coated (1000 rpm for 60 s) on graphene and dried in the vacuum oven at 60°C for 30 mins before transfer.

Following the coating step of the support layer, the graphene film was transferred to the porous substrates (for gas permeation measurement) or the silicon wafer (characterization purposes). Briefly, the Cu foil was etched by floating the graphene/Cu on 1 M FeCl_3_. The film was transferred to a 1 m HCl bath for 1 h and a deionized water bath for 1 h. Subsequently, the graphene samples were collected by macroporous support, polymeric support (PBI), and silicon wafer.

The oxidized graphene on the support was directly exposed to NH_3_ vapor to introduce N functional groups. The oxidized graphene was sealed into a conical flask containing 7 N NH_3_ in methanol. Prior to the reaction, the flask was evacuated to remove air residue. The reaction was carried out at 20°C for 24 h. The resulting samples were placed into a vacuum oven at 25°C for 12 h and then in 1 bar Ar at 150 °C to remove the residual solvent.

### 4.4 Dynamically Thermal Tuning N‐Functionalized Graphene 0D Pores

N‐functionalized graphene membranes were subjected to thermal annealing in a CVD furnace. The temperature was ramped at a controlled rate of 2°C min^−1^ using a programmable controller. Throughout the annealing process, an inert argon (Ar) atmosphere was maintained. Each membrane was annealed at the target temperature for 1 h, then allowed to cool naturally to room temperature before being removed from the furnace. Immediately after annealing, the graphene membranes were transferred to the gas permeation setup for characterization of their gas transport properties. The same membranes underwent the same annealing and measurement procedures at different target temperatures to monitor changes in gas transport performance as a function of annealing temperature. Similarly, N‐functionalized graphene on SiO_2_/Si was annealed with the same procedure above for Raman spectroscopy. Samples for STM and XPS were annealed inside the UHV chamber.

### 4.5 Material Characterization

Raman spectrum was collected by using Renishaw micro‐Raman spectroscopy equipped with a green laser (532 nm, 2.33 eV, ×100 objective). The peak properties of the Raman spectrum were subtracted using background subtraction and Gaussian peak fitting for the *D*, *G*, and *2D* peaks.

The LTSTM was conducted at 4 K temperature using liquid He cryostats. HOPG substrate was used to investigate the surface and functional groups of NH_3_‐treated graphene samples to minimize surface roughness and contamination. The clean HOPG surface was obtained after being exfoliated on the top surface a few times. The HOPG samples were oxidized by O_3_ and NH_3_ treatment using the same procedures as above. STM tips were prepared by commercial Pt/Ir wire (Pt: 90 wt.% and diameter of 0.25 mm; Alfa Aesar). The STM images were analyzed by using the WSXM software [76].

A scanning electron microscope (FEI Teneo) was operated to observe the sample surface morphology and cross‐section. Three samples were prepared and imaged to calculate the average and standard deviation of the film thickness.

X‐ray photoelectron spectroscopy (XPS) measurements were performed using an Axis Supra instrument (Kratos Analytical) on N‐functionalized graphene transferred onto a microporous support. Prior to transfer, the O_3_‐treated graphene samples were spin‐coated with 1.25 wt.% poly(1‐trimethylsilyl‐1‐propyne) (PTMSP) dissolved in toluene at 1000 r.p.m. for 30 s, followed by 2000 r.p.m. for another 30 s. Graphene was then transferred to the support via a wet‐transfer process, which was used for removing the underlying Cu substrate. For N‐functionalization, the porous graphene on a macroporous support was exposed to NH_3_ vapor. The resulting samples were immersed in a toluene bath for 2 h to remove the PTMSP coating, followed by drying under an Ar atmosphere. During XPS measurements, samples were electrically grounded to the sample stage within the ultra‐high vacuum (UHV) chamber. Spectra were acquired using a monochromatic Al Kα X‐ray source (1486.6 eV) with an analyzer pass energy of 20 eV. In situ thermal annealing experiments were conducted inside the UHV chamber, where samples were held at the target temperature for 1 h prior to measurement. All spectra were analyzed using CasaXPS software, with background subtraction by the Shirley method.

### 4.6 Gas Permeation Experiment

The permeation measurements were conducted in a homemade permeation setup (Figure S26). The gas permeation setup was connected with a mass spectrometer (MS, Hiden Analytical, HPR‐20) which was pre‐calibrated within a 5% error for collecting the permeation results. The membrane module and the gas line were inside the oven to maintain a certain testing temperature. The membranes module for membranes on porous PBI support was gas‐tight sealed in an annular membrane model (a quarter‐inch Swagelok VCR fitting) by using Vitron rings. The graphene membranes on the macroporous metal support used metal‐to‐metal leak‐tight seal in a custom Swagelok setup [37]. The pressure of the feed was maintained at 2 bar with 1 bar pressure difference between the feed and permeate side. Argon was used as the sweep gas to carry the gas to the MS. Steady state results were extracted and reported for gas permeation properties of membranes.

The permeance, ***J***_***i***_, for gas, i is given by

(7) $$J_{i}=Q_{i}/\left(A\cdot \Delta P_{i}\right)$$

where ***Q***_***i***_ is the molar flow rate of gas ***i*** across the membrane, **A** is the effective membrane area, and ***ΔP***_***i***_ pressure difference between the feed and permeate sides for the component ***i***. The gas pair selectivity of two gases, ***i*** and ***j***, was calculated by dividing the permeance of gas ***i*** by the permeance of gas ***j***. For the mixture gas permeation tests, the separation factor of two gases, ***i*** and *** j***, was calculated using Equation (8).

(8) $$\alpha_{ij}=\frac{\;{\left(\frac{C_{i}}{C_{j}}\right)}_{\mathrm{permeate}}}{{\left(\frac{C_{i}}{C_{j}}\right)}_{\mathrm{feed}}}\;$$

### 4.7 Statistical Analysis

The reported data with error bars refer to the standard deviation across more than three samples. The center of each error bar represents the average of the measurements from the samples. The STM data was processed by using WSXM software. Analysis of the Raman data was carried out in MATLAB to extract the peak properties. The background from the spectrum was subtracted using the least‐squares curve fitting tool.

## Conflicts of Interest

KVA is a cofounder of a spinoff aiming to commercialize porous graphene membranes for the carbon capture application.

## Supporting information




**Supporting File**: adma71930‐sup‐0001‐SuppMat.docx

## Data Availability

The data that support the findings of this study are available from the corresponding author upon reasonable request.
