# Supplementary material for: Tuning Pore Size in Porous Graphene Membrane for O2/N2 Separation
Source: Adv Mater. 2025 Dec 28;38(10):e19645. doi: 10.1002/adma.202519645 (PMC12910542; doi:10.1002/adma.202519645)
Supplement: Supplementary file 1 — Supporting File: adma71930‐sup‐0001‐SuppMat.docx [file ADMA-38-e19645-s001.docx]

Supporting Information

**Tuning pore size in porous graphene membrane for O_2_/N_2_ separation**

*Kuang-Jung Hsu^1^, Marina Micari^1^, Yueqing Shen^1^, Shaoxian Li^1^, Shuqing Song^1^, Kumar Varoon Agrawal^1^**

^1^Laboratory of Advanced Separations, École Polytechnique Fédérale de Lausanne (EPFL), 1950 Sion, Switzerland

**Supplementary Note 1. The apparent activation of energy for gas transport**

Gas transport across the selective zero-dimensional pores on graphene experiences an activation energy barrier for translocation. The translocation is a temperature-activated transport mechanism which can be described as the temperature-dependent gas flux with an Arrhenius relationship.

$Flux= A_{0}\exp\left( \frac{-E_{app-act}}{RT} \right)$ (1)

where $A_{0}$ combines the pre-exponential factor for the Arrhenius term and the density of gas occupied pores. $E_{app-act}$ is essentially the sum of the activation energy ($E_{act}$) for gas molecules to translocate through the pores and the adsorption energy ($H_{ad}$) of gas molecules on the graphene pore.

**Supplementary Note 2. The mathematical model for estimating selective/non-selective transport and the permeable pore density across the graphene membranes.**

The pore size distribution on graphene membranes is in a typical lognormal distribution. The permeance of graphene membranes results from contributions by both selective and non-selective pores. Understanding the contribution of each pore type to the overall permeance for various gases enables tuning the gas separation performance.

For the non-selective pores, gas transport through non-selective pores primarily follows effusion transport across zero-dimensional pores with steric effects. The effusion transport, as proposed by Wang et al.,^[1]^ is described as

$P_{e}= \frac{1}{\sqrt{2\pi mk_{B}T}}\frac{\pi{(D_{p}-D_{m})}^{2}}{4}$ (2)

where $P_{e}$ is the effusion transport across the zero-dimensional pore, *m* is the gas molecular weight, $k_{B}$ is the Boltzmann constant, *T* is the absolute temperature in degrees Kelvin, $D_{p}$ is the pore diameter, and $D_{m}$ is the diameter of molecules.

However, graphene membranes are supported by a support layer so the gas transport resistance across the membrane is governed by the support layer. The NPC support layer in this work hosts rigid porous channels, allowing the gas transport through the support film with high gas permeance.^[2]^ The permeance of NPC can be expressed as $P_{s}$.

For gas transport through the small zero-dimensional pores, the translocation step is the rate-limiting step.^[3]^ Gas transport in this regime correlates with the activation energy required for translocation and is expressed as:

$P_{act}= \frac{1}{2\sqrt{2\pi mk_{B}T}}\frac{\pi{(D_{p}-D_{m})}^{2}}{4}erfc\sqrt{\frac{E_{app-act}}{k_{B}T}}$ (3)

where erfc is the complementary error function, $E_{app-act}$ is the activation energy for gas transport across the zero-dimensional pores.

Recently, molecular dynamics simulation and transition state theory have been successfully used to predict the gas transport across the functionalized pores through the activated transport (Table 2, Supporting Information).^[4]^ The selective Å-scale pores can be categorized into two kinds: (1) small, highly selective pores where transport requires high *E*_act-app_ leading to lower permeance ($P_{sel, s}$). (2) larger, moderately selective pores with faster transport across pores requiring a lower *E*_act-app_ for translocation, leading to higher permeance ($P_{sel, m})$. The total gas permeance from selective pores ($P_{T, sel}$) can be obtained by summing transport from all selective pores, using the number density of pores ($\rho_{sel, s}$ and $\rho_{sel, m}$, unit of cm^-2^).

$P_{T, sel}= {\rho_{sel, s}P}_{sel, s}+ {\rho_{sel, m}P}_{sel, m}$(4)

For NPC-supported graphene membranes, the total permeance is a combination of selective and non-selective pore contributions. Assuming, one has a fractional area ($\theta_{nonsel}$) as nonselective, one gets effective permeance of nonselective part of the membrane as

$P_{T,nonsel}= {\theta_{nonsel} P}_{support}$ (5)

where $\theta_{nonsel}$ varies between 0 and 1, with 0 referring to perfectly selective membrane and 1 referring to a perfectly nonselective membrane. The total permeance is then

$P_{T} = {\rho_{sel, s}P}_{sel, s}+ {\rho_{sel, m}P}_{sel, m} + {\theta_{nonsel} P}_{support}$ (6)

Given that the O_3_-derived pore size distribution is lognormal and contains a small fraction of non-selective pores, which are similar for O_2_ and N_2_, we assume that $\theta_{nonsel}$​ is approximately the same for all gases. By substituting the O_2_ and N_2_ permeability coefficients (Table S2, Supporting Information) into equation S6, Supplementary Information and regressing against the measured permeance values, we determined $\rho_{sel, s}$, $\rho_{sel, m}$ and $\theta_{nonsel}$ for membranes annealed at 150 and 300 °C. The resulting $\theta_{nonsel}$ values indicate that the fraction of non-selective pores is only a few ppm (Figure S17, Supporting Information).

This mathematical model provides a framework for estimating the permeance contributions from selective and non-selective pores in graphene membranes, accounting for the effects of pore size distribution, permeable pore density, and fraction of selective transport and non-selective transport.

We also used a model for calculating the pore diameter ($D_{p}$​) and the apparent activation energy for gas transport ($E_{act}$) based on gas permeation results.

- The apparent activation energy for gas transport

The activated gas transport through the graphene pores is a temperature-dependent transport mechanism. The apparent activation energy for gas transport, $E_{act}$, can be extracted by fitting the temperature-dependent gas permeance to the Arrhenius equation (equation S1, Supplementary Information)

- Estimating the pore diameter

The pore diameter ($D_{p}$​) can be estimated using the measured selectivity of gas molecules​, modeled by the steric transport mechanism.^[1,5]^ In this model, the effective pore area ($A_{eff}$​) and total pore area ($A_{p}$) are defined as:

$A_{eff}= \frac{\pi}{4}(D_{p}-D_{m})^{2}$ (7)

$A_{p}= \frac{\pi}{4}D_{p}^{2}$ (8)

The transmission probability ($T_{prob}$​) is given by:

$T_{prob}= \frac{J_{gas}}{J_{gas-ideal}}= \frac{A_{eff}}{A_{p}}$ (9)

where $J_{gas}$ is the actual gas flux across the pores, on the other hand, $J_{gas-ideal}$ represents the ideal gas flux across the pores.

$J_{gas-ideal}= \frac{\Delta p}{\sqrt{2\pi mk_{B}T}}$ (10)

where $\Delta p$ is the pressure difference between the feed and permeate side.

By dividing the transmission probabilities of two gases, the ratio of effective pore sizes can be determined:

$\frac{T_{prob, 1}}{T_{prob, 2}}= \frac{A_{eff, 1}}{A_{eff, 2}}=\frac{J_{gas, 1}}{J_{gas,2}} \frac{J_{gas-ideal, 2}}{J_{gas-ideal,1}}$ (11)

Expressing selectivity $S\left( \frac{gas,1}{gas,2} \right)$ as the ratio of $\frac{J_{gas, 1}}{J_{gas,2}}$ ​, the equation can be expressed as:

$\frac{A_{eff, 1}}{A_{eff, 2}}=S\left( \frac{gas,1}{gas,2} \right)\frac{\sqrt{2\pi m_{gas,1}k_{B}T}}{\sqrt{2\pi m_{gas,2}k_{B}T}}=S\left( \frac{gas,1}{gas,2} \right)\frac{\sqrt{m_{gas,1}}}{\sqrt{m_{gas,2}}}$ (12)

Substituting $A_{eff}= \frac{\pi}{4}(D_{p}-D_{m})^{2}$, we derive:

$\frac{(D_{p}-D_{m,1})^{2}}{(D_{p}-D_{m,2})^{2}}=S\left( \frac{gas,1}{gas,2} \right)\frac{\sqrt{m_{gas,1}}}{\sqrt{m_{gas,2}}}$ (13)

where $D_{m,1}$ and $D_{m,2}$ are the kinetic diameters of gas 1 and 2.

Rearranging yields an expression for $D_{p}$​,

$D_{p}= \frac{D_{m,1}-\sqrt{S\left( \frac{gas,1}{gas,2} \right)}{(\frac{m_{gas,1}}{m_{gas,2}})}^{0.25}D_{m,2}}{1-\sqrt{S\left( \frac{gas,1}{gas,2} \right)}{(\frac{m_{gas,1}}{m_{gas,2}})}^{0.25}}$ (14)

Using the selectivity of He and N_2_​ from the gas permeation results, we can estimate the corresponding pore diameter $D_{p}$.

**Supplementary Note 3. Tuning porosity of porous graphene membranes via controlled ozone oxidation**

As-synthesized chemical vapor deposition (CVD) graphene membranes were introduced into a millisecond ozone (O_3_) reactor to generate angstrom-scale pores.^[6]^ The resulting porous graphene hosts a log-normal pore size distribution, with a high density of non-permeable pores.^[6,7]^ To enhance gas permeance, the porous graphene on Cu substrates were exposed to continuous O_3_ flow at 20 °C for varying durations to expand pores. Longer O_3_ exposure times led to larger pore size and increased porosity.^[6,7]^ In this work, we define graphene exposed to 30 minutes of O_3_ etching as graphene with mild oxidation, and that exposed to 60 minutes as graphene with high oxidation. After pore expansion, the membranes were further functionalized with NH_3_ to yield N-functionalized porous graphene. The graphene with high oxidation had a molecular cut-off between O_2_ and N_2_ (Figure 2c), while the mild-oxidation sample had a cut-off between CO_2_ and O_2_ (Figure S18, Supporting Information). These differences are primarily attributed to different levels of pore expansion, resulting in an increased number of permeable pores and different pore size distributions.

The electron density gap within N-functionalized zero-dimensional pores can be dynamically tuned through post-thermal annealing. This annealing process also may induce the gasification of certain oxygen-containing functional groups, such as epoxy and ether groups, on the graphene lattice.^[5]^ In highly porous graphene, this may result in undesired pore coalescence. Porous graphene prepared under mild oxidation conditions exhibits lower porosity, making it a more suitable model system for investigating the influence of thermal annealing on adjusting the size of the electron density gap.

**Supplementary Note 4. Techno-economic analysis for membrane processes**

*Technical model*

This work present techno-economic results relevant to a single membrane stage with cross-flow arrangement. To model the single stage, we used the following assumptions: steady-state operation, isothermal process, negligible pressure drops and concentration polarization.

The membrane unit is discretized along the length (z axis) into a certain number of elements (N) with membrane area *dA* [m^2^]. For any discretization element, the transmembrane flux of each component (J_i_ [mol m^-2^s^-1^]) is calculated as function of the partial pressure difference across the membrane and the permeance of the component (**P**_i_ [mol m^-2^s^-1^Pa^-1^]). The partial pressure depends on the molar fractions ($x_{f,i}$ and $x_{p,i}$) and the total pressures on the feed and permeate side.

| $J_{i}=\boldsymbol{P}_{i} \left( P_{feed} x_{f,i}-P_{perm} x_{p,i} \right)$ | (17) |
| --- | --- |

The driving force can be generated via compressing the feed (higher $P_{feed}$) or evacuating the permeate channel (lower $P_{perm}$) or a combination of the two. In this work, we always consider P_feed_ of 1 bar and vacuum pressures (minimum of 0.1 bar) in the permeate channel.

The total flux is given by the sum of the fluxes of each component and is used for mass balances to generate the profiles of total flow rates on the retentate ($Q_{r}$) and permeate ($Q_{p}$) channels (Equation S18 and S20, Supplementary Information). Similarly, the fluxes of each component are used to estimate profiles of concentration ($x_{r,i}$ and $x_{p,i}$) along the length of the module (Equation S19 and S21, Supplementary Information). Importantly, the feed flow rate and composition of each element is defined as equal to the flow rate and composition of the retentate produced by the previous element (Equation S22 and S23, Supplementary Information).

| $Q_{r}\left( z \right)=Q_{f}\left( z \right)- \sum_{i} J_{i}\left( z \right) dA$ | (18) |
| --- | --- |
| $Q_{r}\left( z \right) x_{r,i}\left( z \right)=Q_{f}\left( z \right) x_{f,i}\left( z \right)- J_{i}\left( z \right) dA$ | (19) |
| $Q_{p}\left( z \right)= \sum_{i} J_{i}\left( z \right) dA$ | (20) |
| $Q_{p}\left( z \right) x_{p,i}\left( z \right)= J_{i}\left( z \right) dA$ | (21) |
| $Q_{f}\left( z+\Delta z \right)= Q_{r} \left( z \right)$ | (22) |
| $x_{f,i}\left( z+\Delta z \right)= x_{r,i} \left( z \right)$ | (23) |

The performance targets are recovery and purity of the main component, i.e., oxygen, as defined in Equation S24 and S25, Supplementary Information.

| $Purity= \frac{\sum_{N} Q_{p}\left( z \right)x_{p,O2}\left( z \right)}{\sum_{N} Q_{p}\left( z \right)}$ | (24) |
| --- | --- |
| $Recovery= \frac{\sum_{N} Q_{p}\left( z \right)x_{p,O2}\left( z \right)}{Q_{feed}x_{feed,O2}}$ | (25) |

*Economic model*

The economic model calculates capital and operating costs based on the outputs (i.e., membrane area and operating pressures) from the technical model.^[8]^

The capital costs are composed of the costs for membranes (specific cost of 100 $ m^-2^, lifetime of 5 years) and for vacuum pumps (lifetime of 25 years). The cost of vacuum pumps is calculated via the six-tenths rule (Equation S26, Supplementary Information) by taking as a reference a vacuum pump with known capacity (expressed as suction volumetric flow rate ($Q_{in,ref}^{vol}$ [m^3^ h^-1^])) and cost (C_VP,ref_ [$]).

| $C_{VP}=C_{VP,ref}\left( \frac{Q_{in}^{vol}}{Q_{in,ref}^{vol}} \right)^{0.6}$ | (26) |
| --- | --- |

The operating costs are composed of the energy cost and the maintenance cost. The first depends on the power requirement in the vacuum pumps (P_VP_ [kW]), calculated as in Equation S27, Supplementary Information as function of the efficiency of the vacuum pump (η_VP_ equal to 60%), the specific heat ratio (*k* [-]), the inlet molar flow rate (Q_in_ [mol s^-1^]), the operating temperature (T [K]) and the suction and the discharge pressures (P_in_ [Pa] and P_out_ [Pa]). The energy cost is calculated as the product of the total power, the specific electricity cost (0.06 $ kWh^-1^) and the annual operating hours (8000 h y^-1^).

| $P_{VP}=\frac{1}{\eta_{VP}}\frac{k}{k-1}Q_{in}RT\left( \left( \frac{P_{out}}{P_{in}} \right)^{\frac{k-1}{k}}-1 \right)$ | (27) |
| --- | --- |

Finally, the cost for maintenance is calculated as the sum of 3.6%/y of the investment cost for pumps and 1%/y of the investment cost for membranes.

The economic performance indicator is the specific cost of equivalent pure oxygen ($/ton_EPO2_). The molar flow rate of EPO2 corresponds to the moles of pure oxygen, n_EPO2_, that should be added to air (n_air_) to achieve the O_2_ purity of the OEA, x_OEA,O2_, as per Equation S28, Supplementary Information.

| $\frac{n_{EPO2}}{n_{OEA}}= \frac{n_{EPO2}}{n_{EPO2}+n_{air}}=\frac{x_{OEA,O2}-0.21}{0.79}$ | (28) |
| --- | --- |

**Supplementary Note 5. Case study on natural gas combustion with Oxygen Enriched Air (OEA)**

In the manuscript, we discuss the energy and cost benefit of applying the proposed membranes to produce OEA for natural gas combustion. For the case study, we consider a furnace operating with flue gas temperature of 1649 °C and without waste heat recovery. The furnace operating with air has a firing rate of 293 kWh (1MMBtu) and requires 23.8 m^3^ of natural gas (1000 scf). This requirement reduces by 45% and 60% when using OEA with purity between 30% and 50%.^[9]^

For the net fuel saving, we calculate the fuel required to provide electricity to membrane process by considering a heating value of natural gas equal to 36.6 MJ/m^3^ and a conversion efficiency of 0.3. Therefore, the net energy and cost benefits consider the avoided fuel given by the higher combustion efficiency and the additional energy requirement and cost for OEA production. For cost calculation, we take the cost of natural gas equal to 0.17 $/m^3^ and we calculate the amount of O_2_ required for combustion by considering an excess of 5% with respect to stoichiometric condition.

**Supplementary Note 6. Resistance model for gas transport**

Gas permeance through graphene membranes is governed by the combined resistance of the support layer (NPC or PDMS), the graphene layer, and the porous PBI substrate containing 20 nm pores. Because the resistance of the PBI substrate is negligible, the total resistance can be written as

R_T_ = R_support_+R_graphene_ (29)

where R_support_ and R_graphene_ represent the transport resistance of the support and graphene selective layers, respectively.

Since permeance is the reciprocal of resistance, the permeance from the resistance model is expressed as^[10]^

$\frac{1}{J_{total}}=\frac{1}{J_{support}}+\frac{1}{J_{graphene}}$ (30)

where $J_{total}$, $J_{support}$, and $J_{graphene}$ are the total permeance of graphene membrane, permeance of standalone support layer, and permeance of standalone graphene layer, respectively.

Given that the permeance of NPC is extremely large ( > 10^5^ GPU), the total permeance of graphene/NPC membrane is close to the permeance of standalone graphene layer, which an average O_2_ permeance of 1870 GPU. By taking the permeance of standalone graphene layer and PDMS layer (11000 GPU of O_2_ permeance), the total permeance of the graphene/PDMS composite membrane is estimated to be approximately 1600 GPU, which is similar to the experimental data.


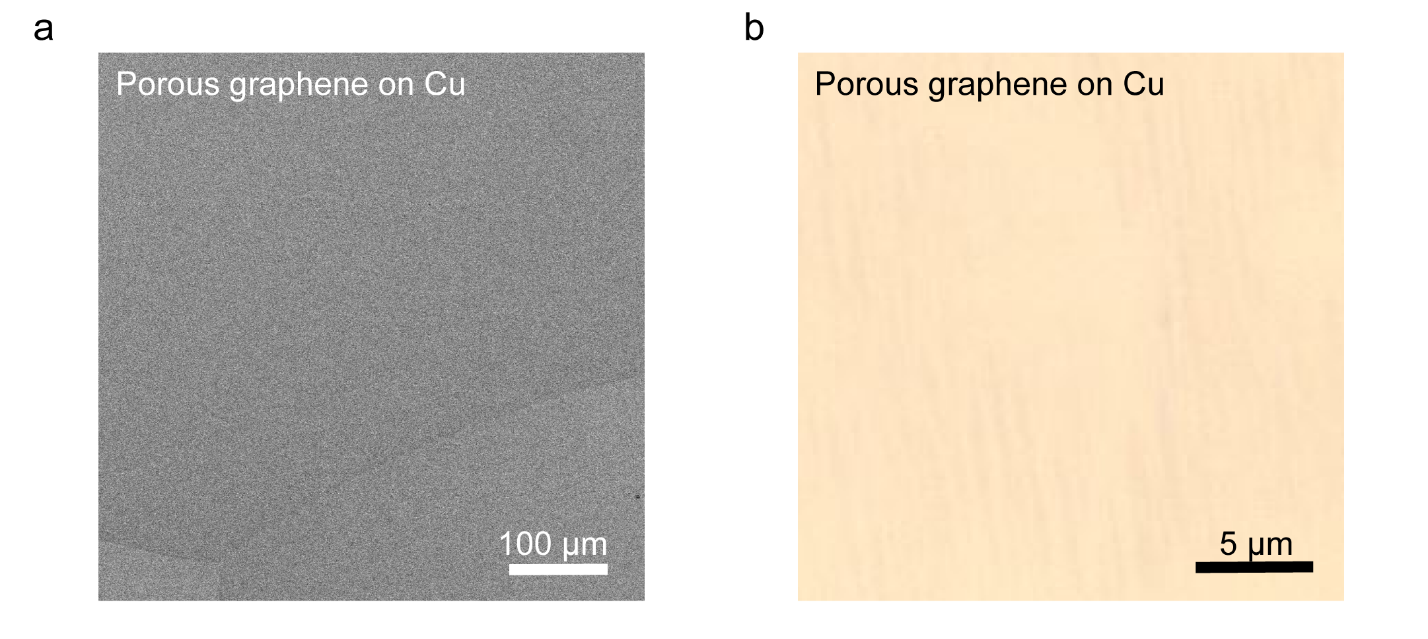


**Figure S1**. The SEM (a) and optical image (b) of porous graphene after O_3_ oxidation and H_2_ reduction at 500 ^o^C.


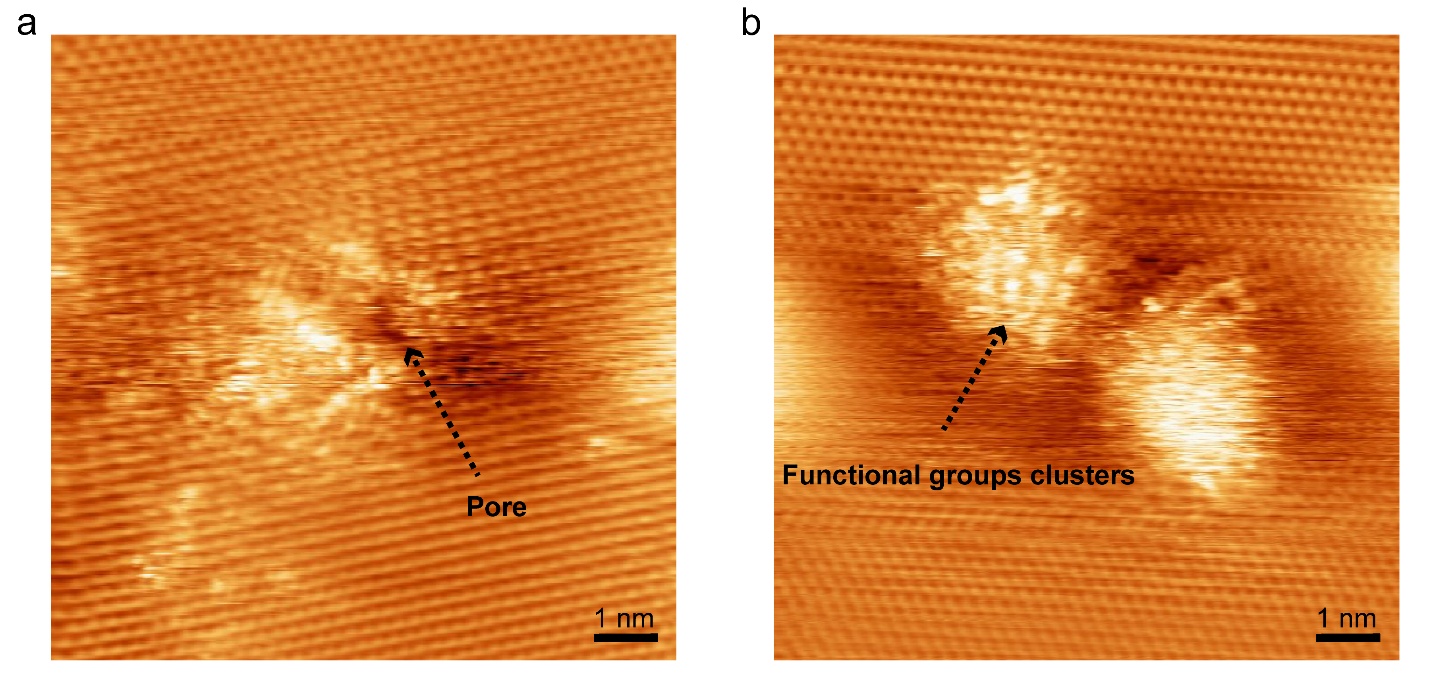


**Figure S2**. The STM image of graphene zero-dimensional pores partially covered (a) or fully covered (b) by N-functional groups (Primary amine, pyridinic N, and their CO_2_-adsorbed derivatives).


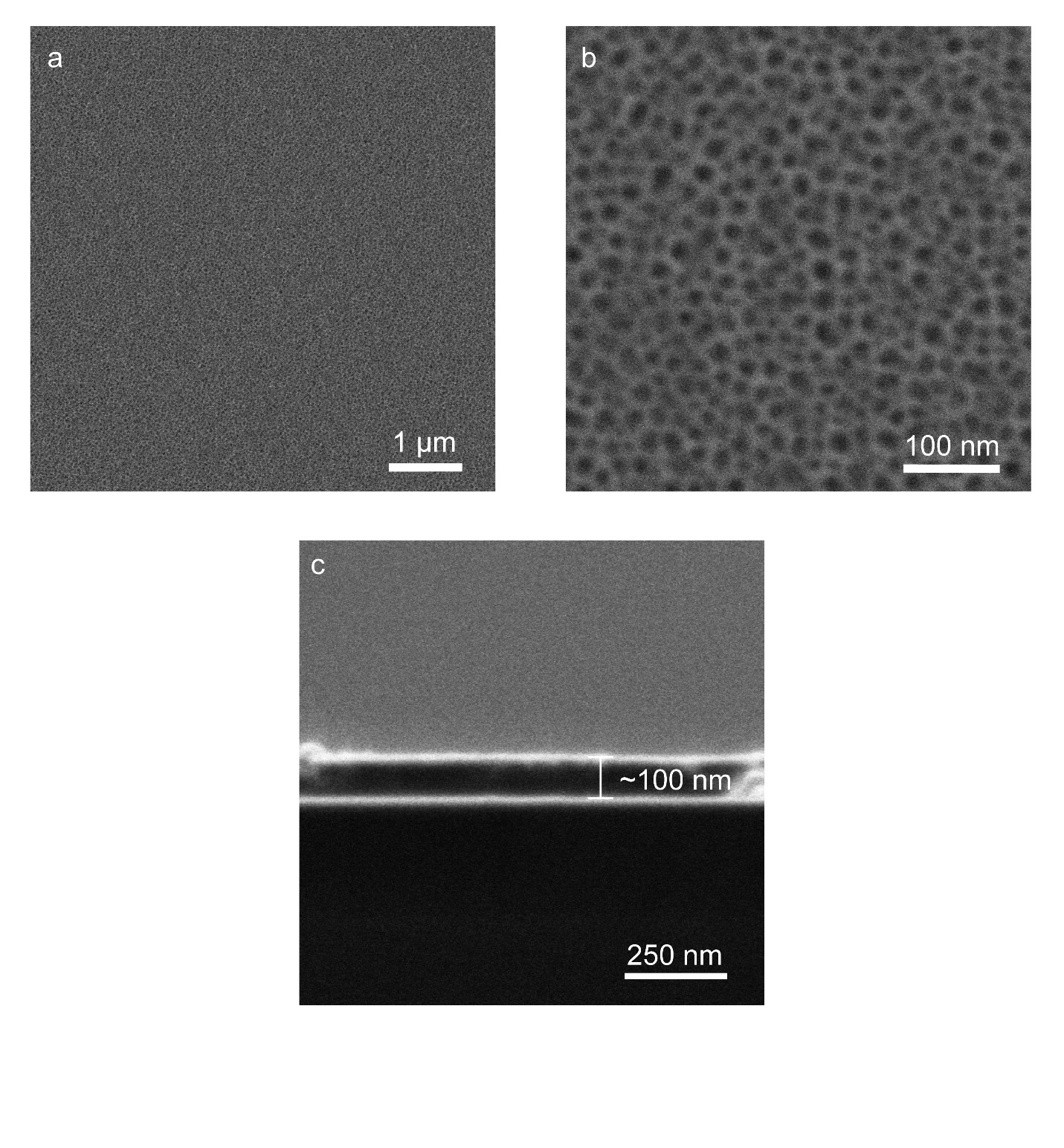


**Figure S3**. SEM characterization and gas measurement of the stand-alone NPC film. SEM images of the surface of NPC film in (a) lower magnification and (b) higher magnification. (c) SEM image of the cross-section of NPC film deposited on a silicon wafer.


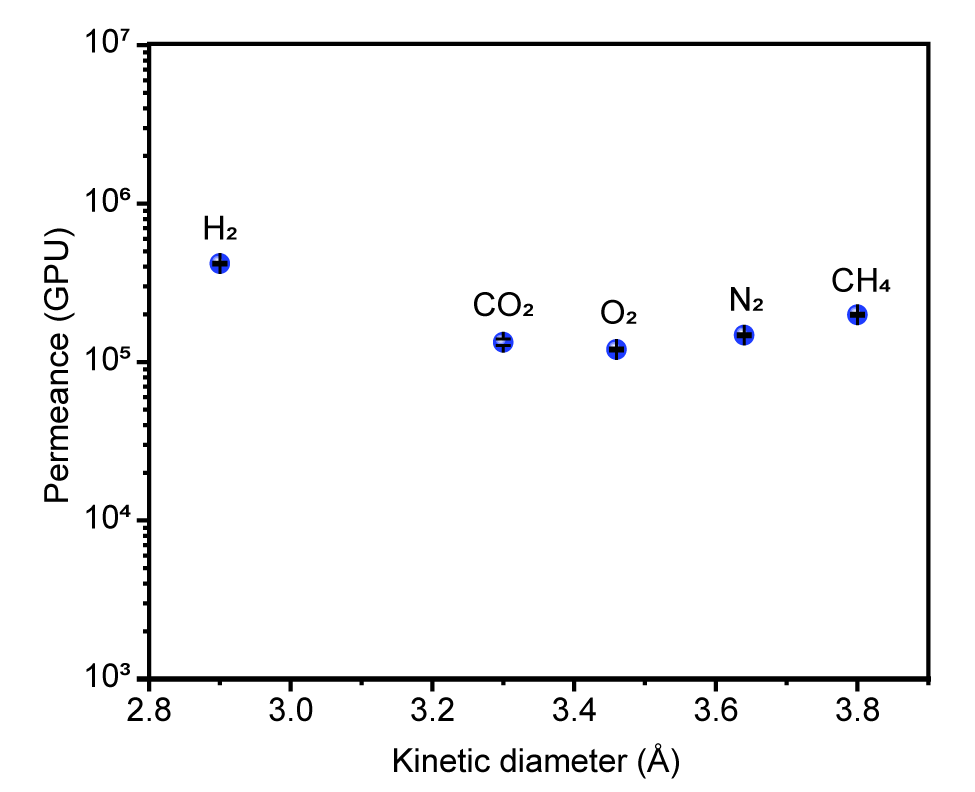


**Figure S4**. Single-gas permeation results of stand-alone NPC membranes measured at 30 ºC, 2 bar. The error bars refer to the standard deviation in the permeance across three membranes. The center of each error bar represents the average separation factor and permeance calculated from the stand-alone NPC membranes.


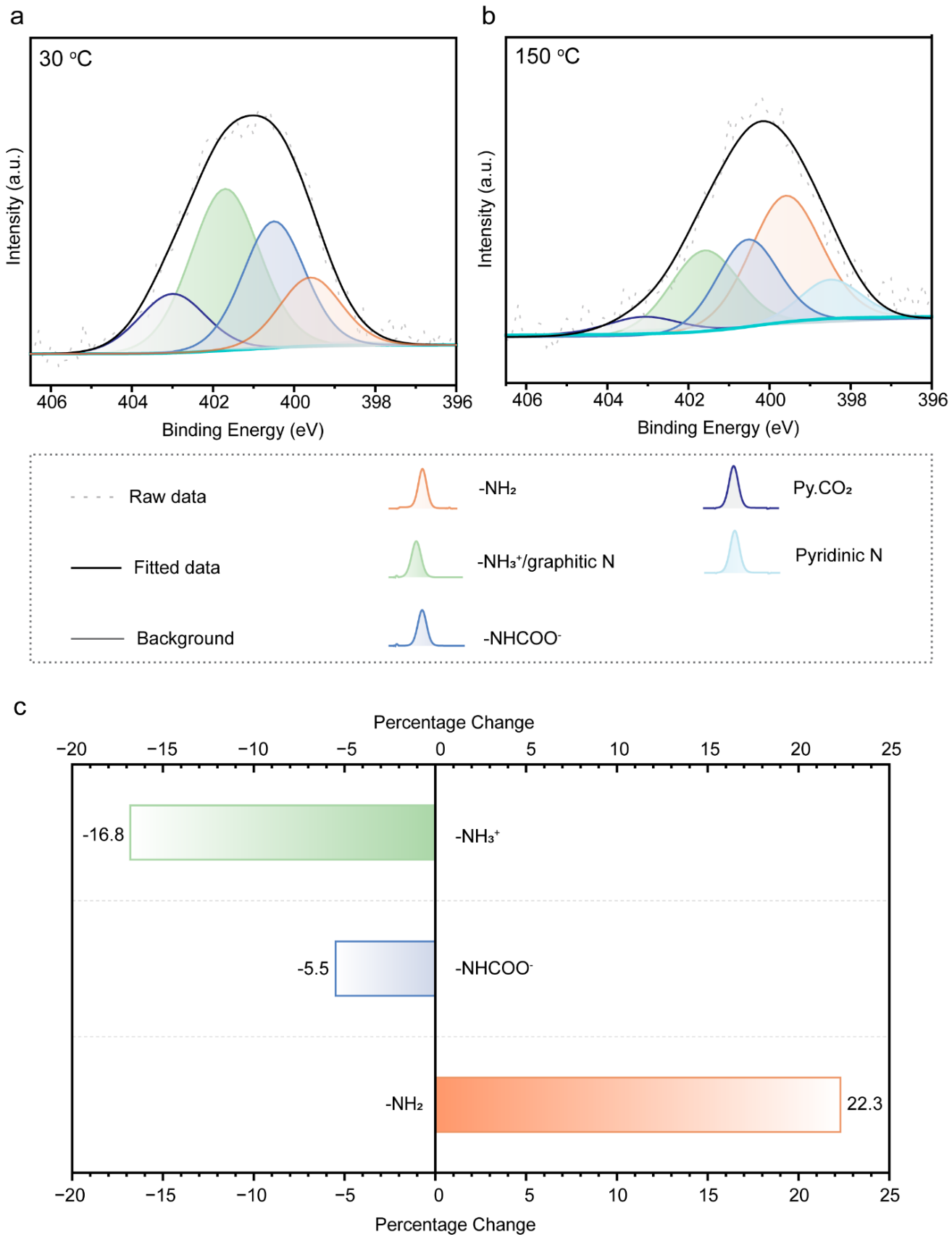


**Figure S5.** Deconvoluted N 1s XPS spectra of N-functionalized graphene measured at 30 °C (a) and 150 °C (b) inside the UHV chamber. (c) Quantification of amine group population differences between 30 °C and 150 °C, showing a decrease due to CO_2_ desorption at elevated temperatures.


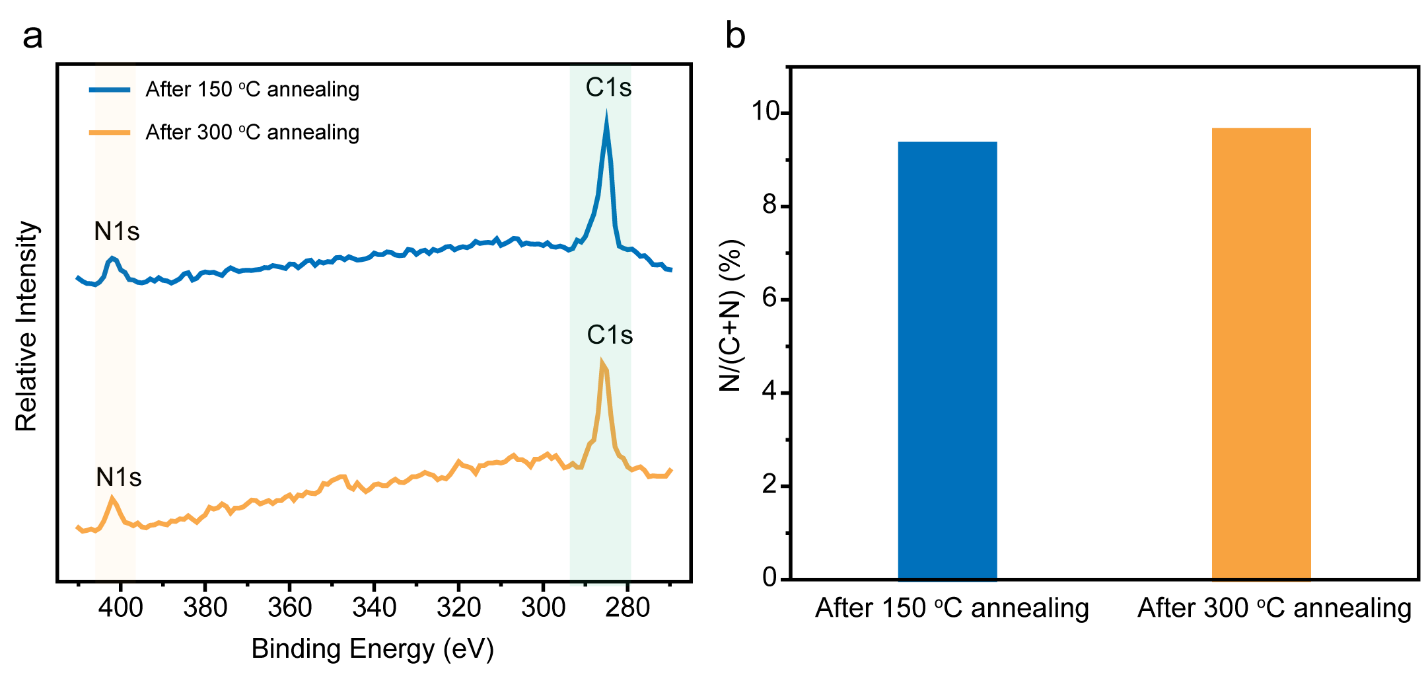


**Figure S6**. XPS analysis of N-functionalized graphene after thermal annealing*.*
**(a)** XPS spectra of N-functionalized graphene after annealing at 150 °C and 300 °C.
**(b)** Nitrogen atomic percentage relative to the total carbon and nitrogen content, extracted from the XPS spectra (a).


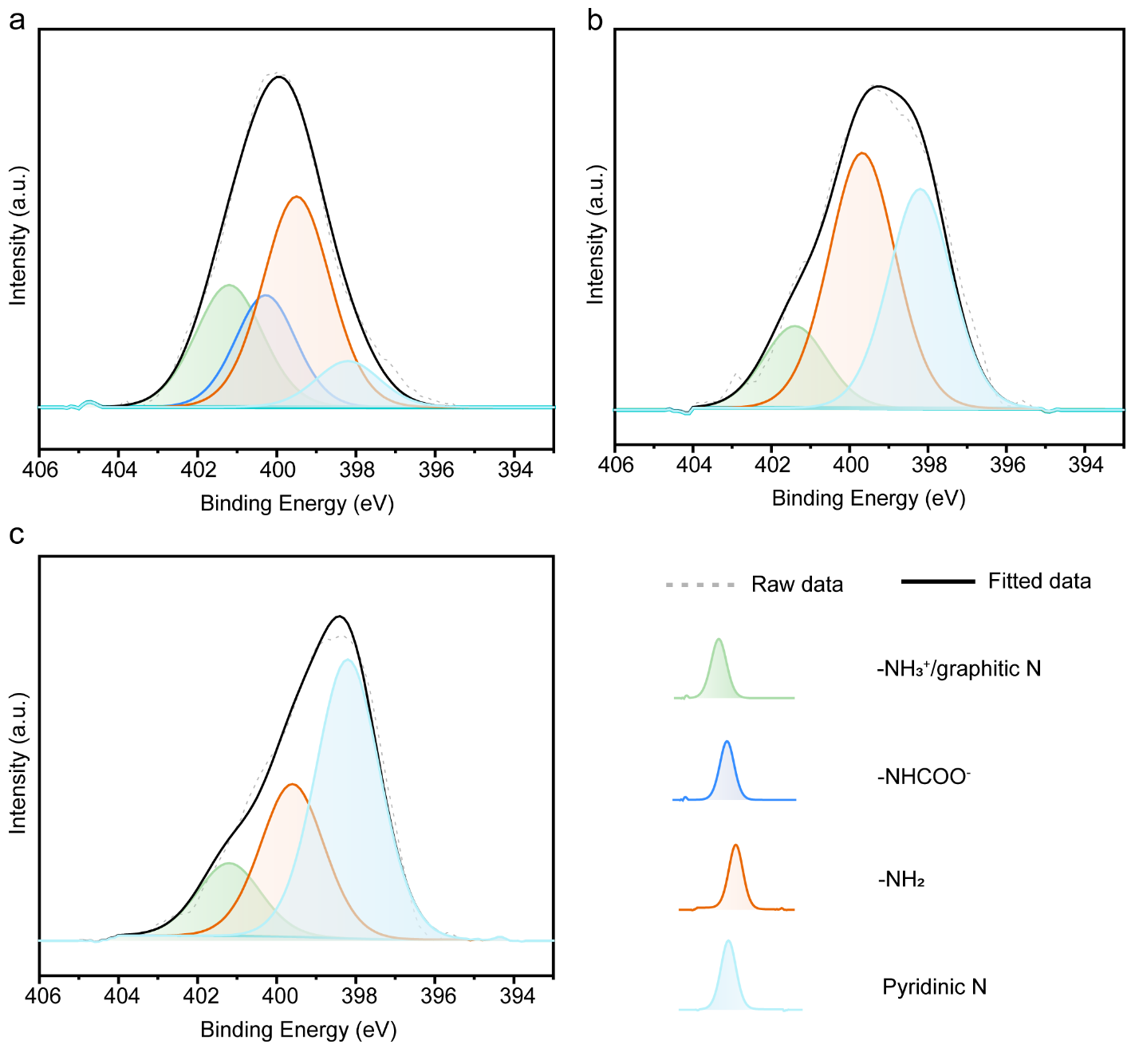


**Figure S7**. N1s XPS spectra and the deconvolution of N-functionalized graphene at various temperatures inside the XPS chamber. (a) 150 ºC, (b) 300 ºC, (c) 400 ºC. The thermal heating was conducted in the UHV chamber for 1h at each temperature.^[11]^


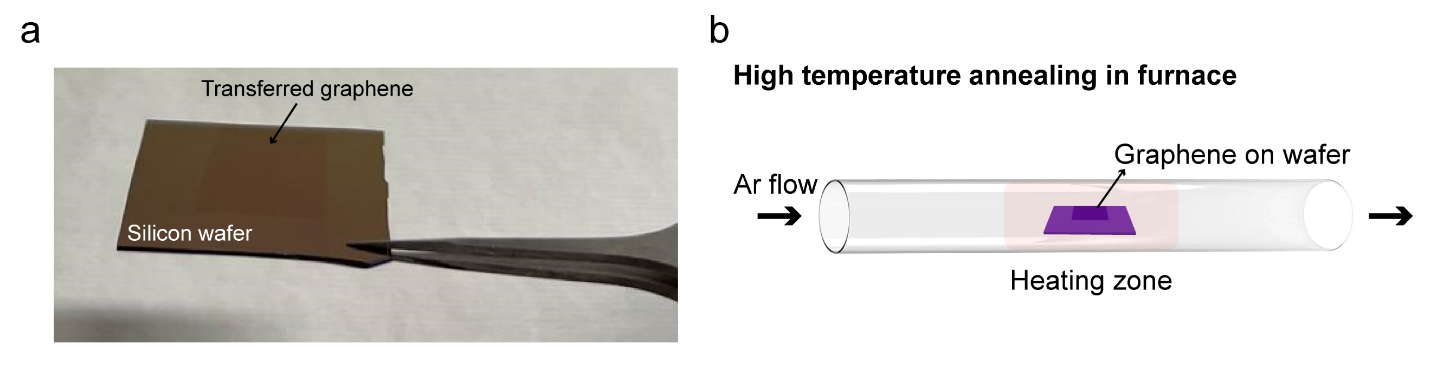


**Figure S8**. (a) Image of transferred graphene (darker contrast) on a silicon wafer. (b) Schematic illustration of the graphene/silicon wafer sample placed inside a furnace for thermal annealing under an Ar atmosphere at 2 bar.


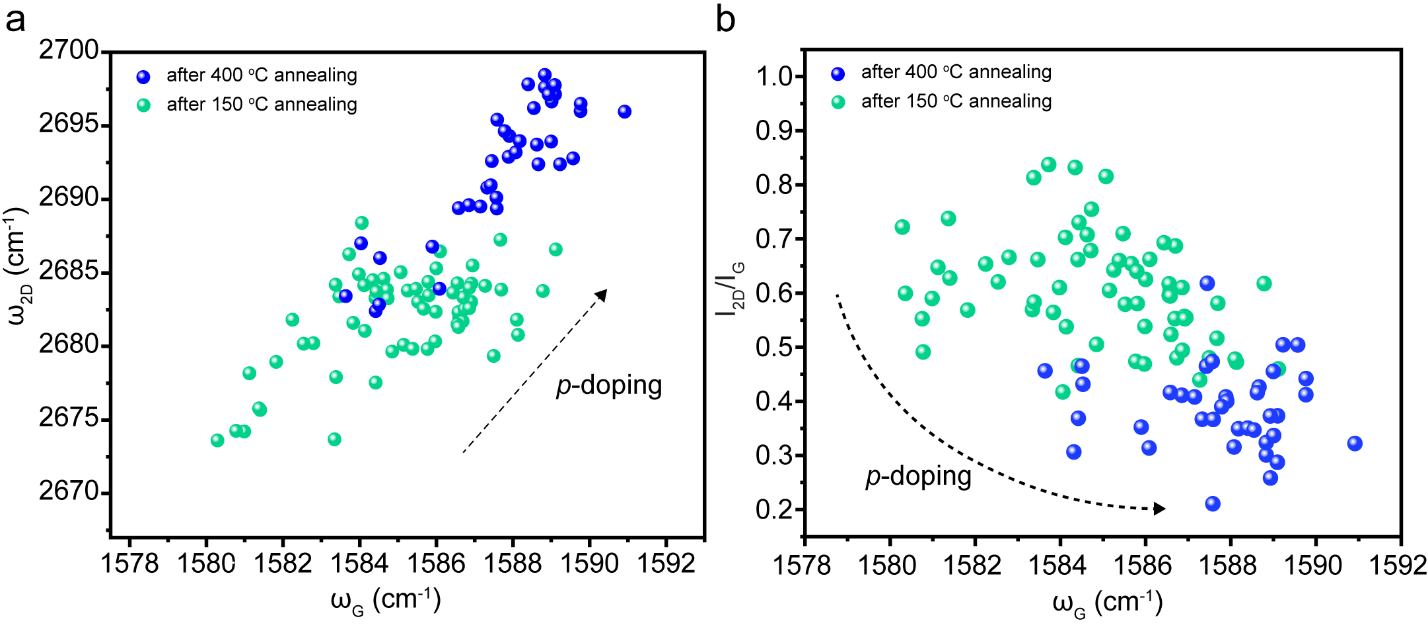


**Figure S9**. Raman spectrum results in understanding the doping level of graphene after various annealing temperatures. (a) *2D* peak position (ω_2D_) versus G peak position (ω_G_) and (b) *I*_2D_/*I*_G_ versus ω_G_. The dotted lines with arrows guide the change of the Raman spectrum with doping level.


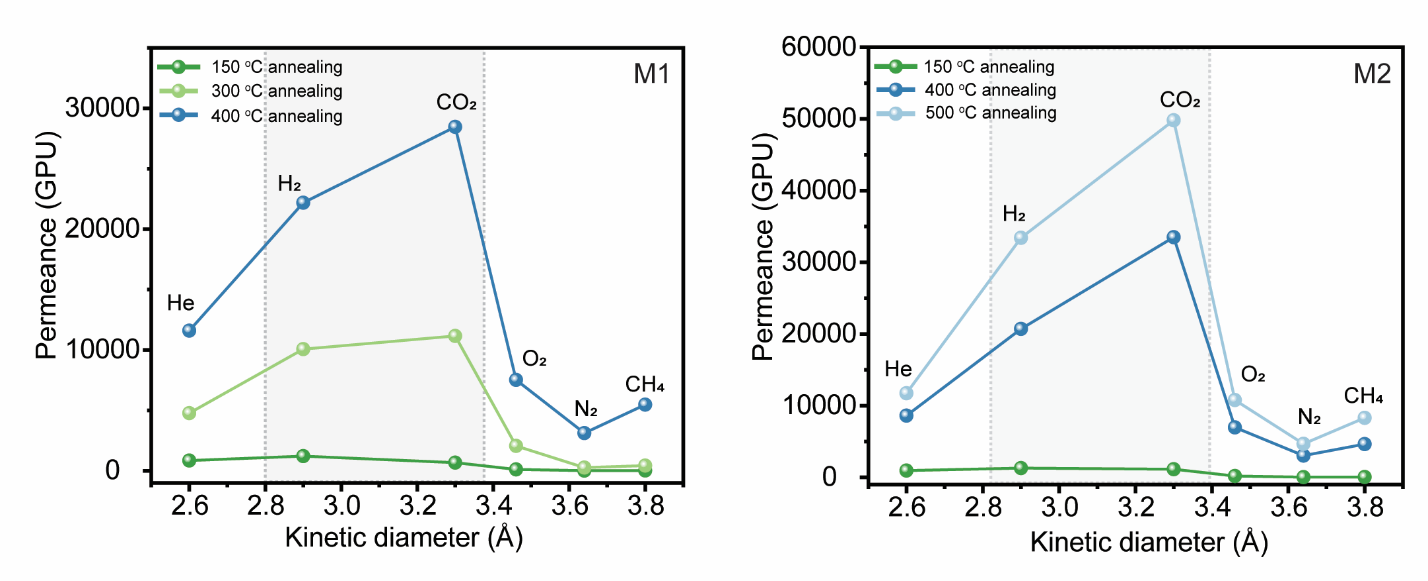


**Figure S10**. Single-gas permeation results of N-functionalized graphene/NPC membranes measured at 30 ºC and 2 bar. The graphene membranes, prepared via mild oxidation, were annealed at 150, 300, and 400 ºC (M1) or 150, 400, and 500 ºC (M2) for 1 h prior to each measurement.


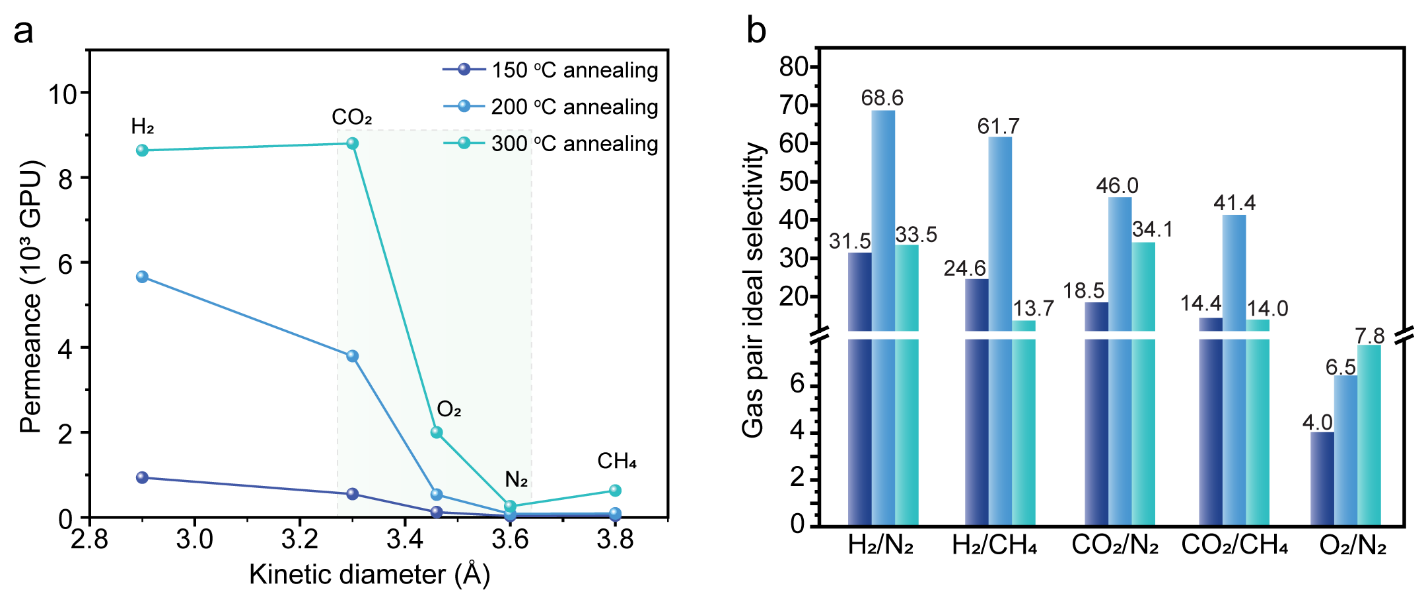


**Figure S11**. Single-gas permeation results of N-functionalized graphene/NPC membranes measured at 30 ºC and 2 bar. (a) Permeance of H_2_, CO_2_, O_2_, N_2,_ and CH_4_, and (b) ideal gas pair selectivity. The graphene membrane, prepared via mild oxidation, was annealed at 150, 200, and 300 ºC for 1h prior to each measurement.


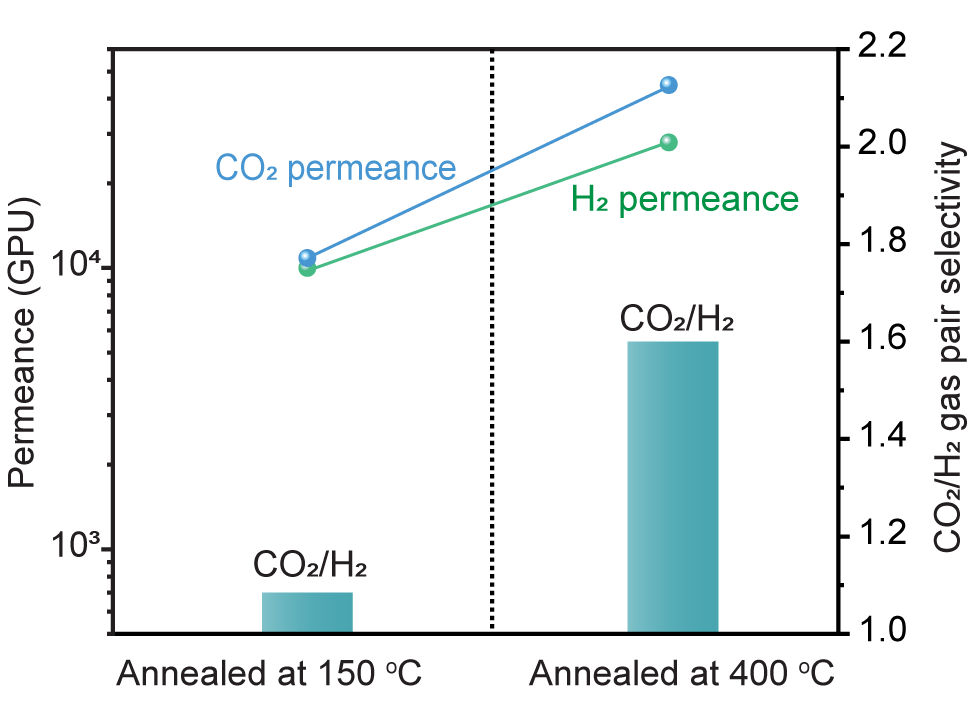


**Figure S12**. Evolution of CO_2_ and H_2_ permeance, as well as CO_2_/H_2_ selectivity, after annealing at 150 ºC and 400 ºC for 1 hour under an Ar environment. After annealing at 400 ºC, CO_2_ transport surpassed H_2_ transport.


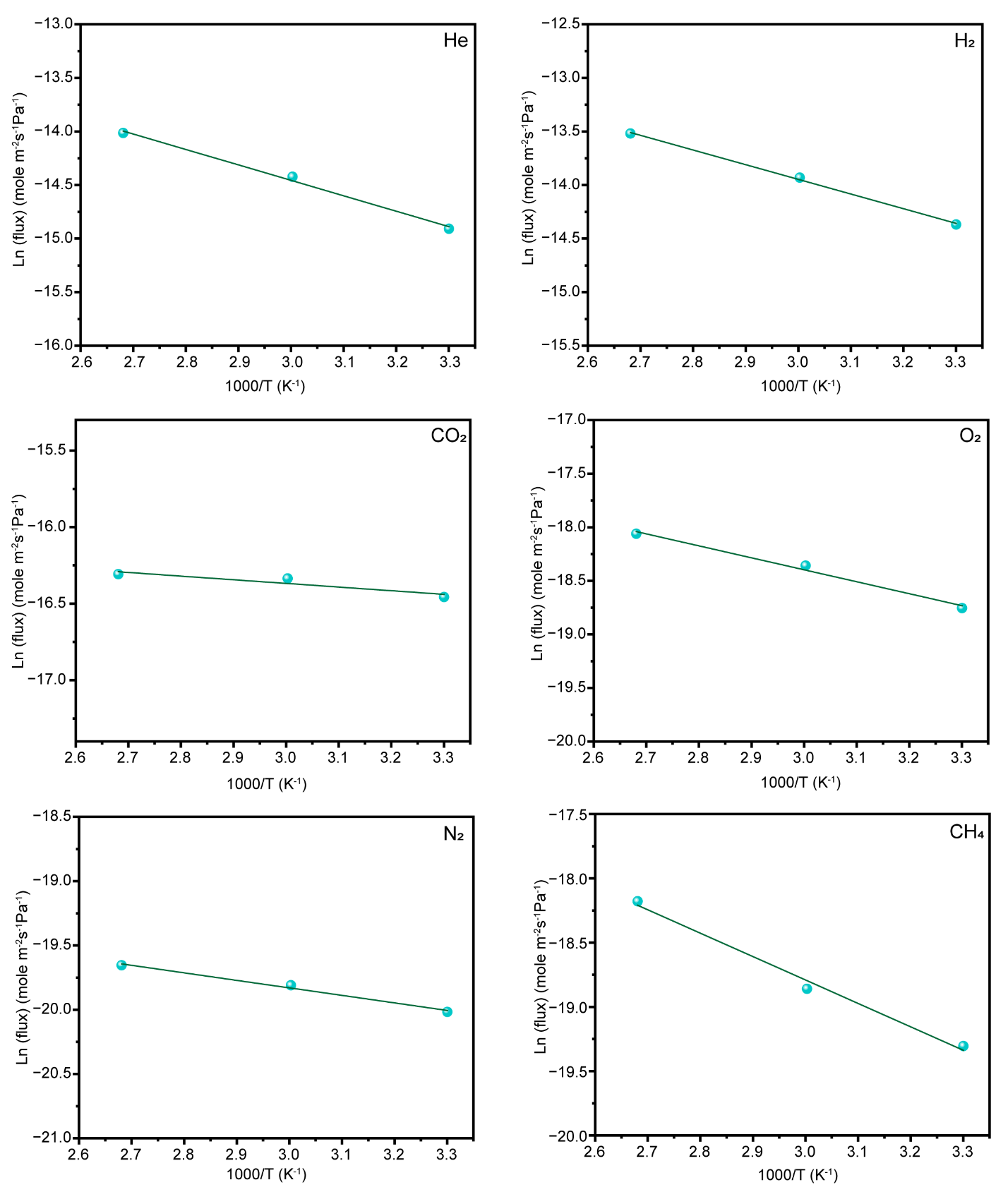


**Figure S13**. A linear fitting plot of the single gas permeation versus reciprocal measurement temperature for apparent activation energy. The data were calculated and measured based on graphene/NPC membranes after 150 ^o^C annealing.


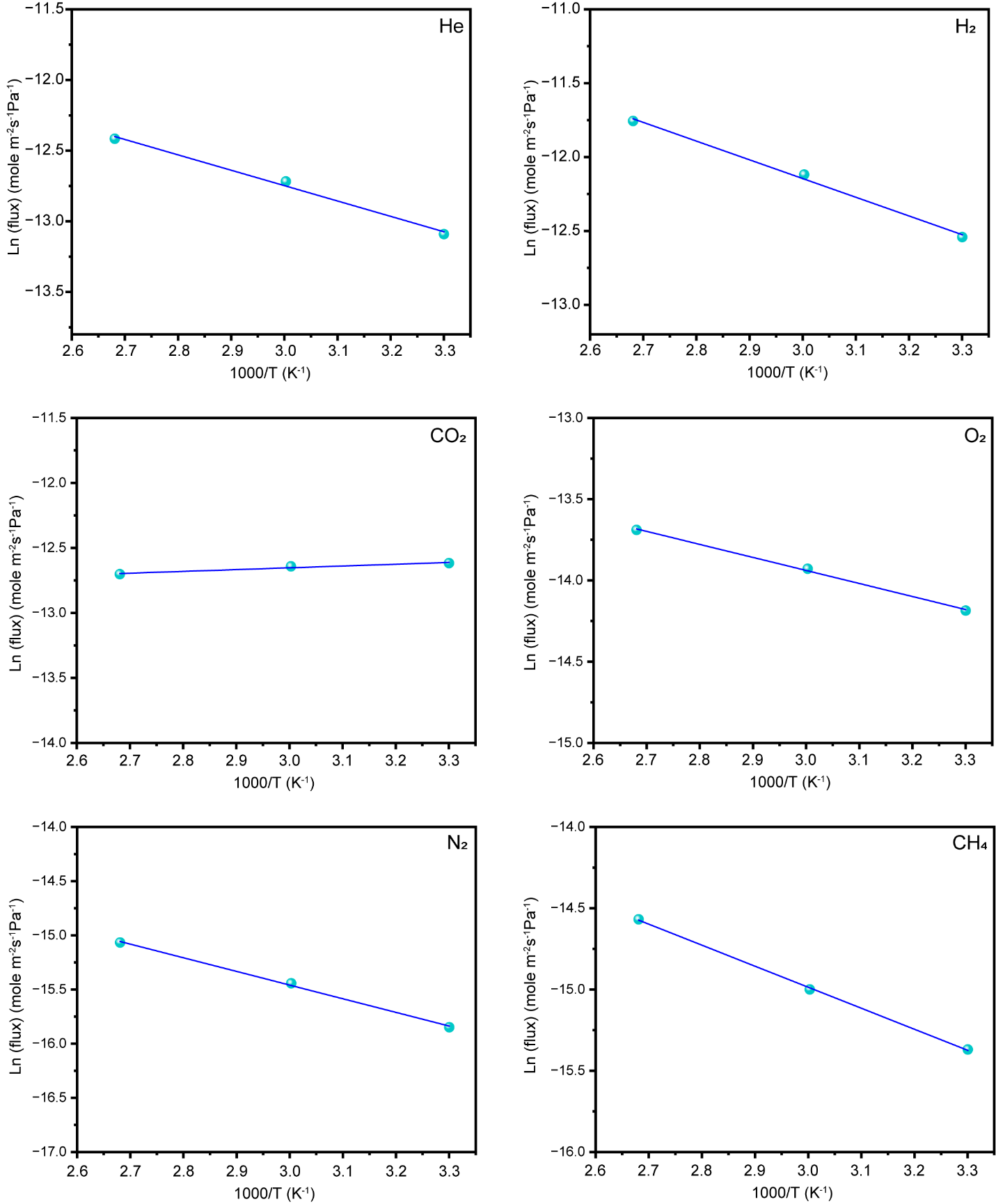


**Figure S14**. A linear fitting plot of the single gas permeation versus reciprocal measurement temperature for apparent activation energy. The data were calculated and measured based on graphene/NPC membranes after 300 ^o^C annealing.


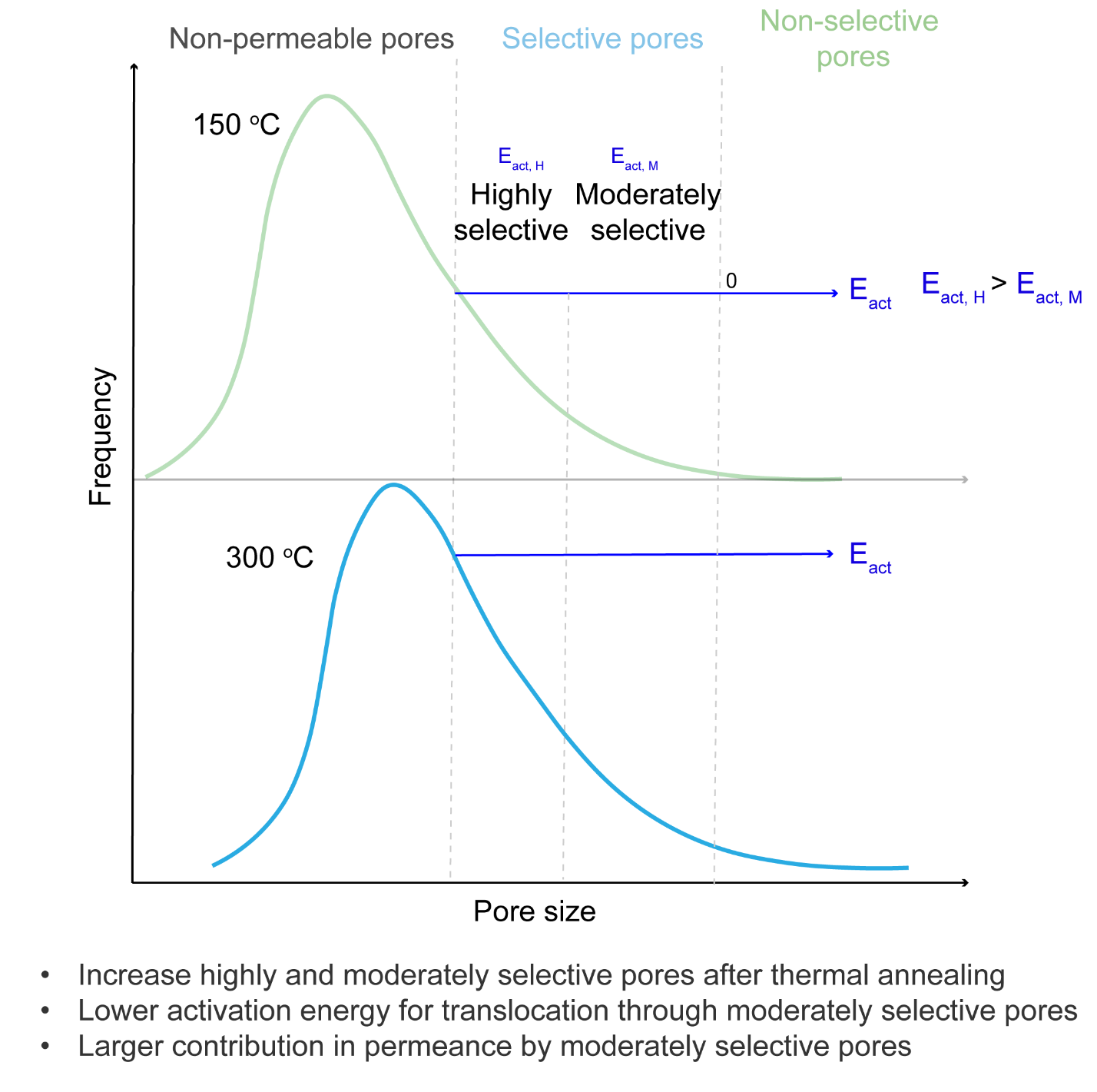


**Figure S15**. Schematic of effective pore size distribution after thermal annealing at 150 and 300 ^o^C. Selective pores are classified into two types: (1) highly selective pores, requiring high activation energy for both O_2_ and N_2_ translocation, and (2) moderately selective pores, where O_2_ translocation requires lower activation energy than N_2_. The latter dominates the total selective transport, contributing the major fraction of permeance (see Figure S16b).


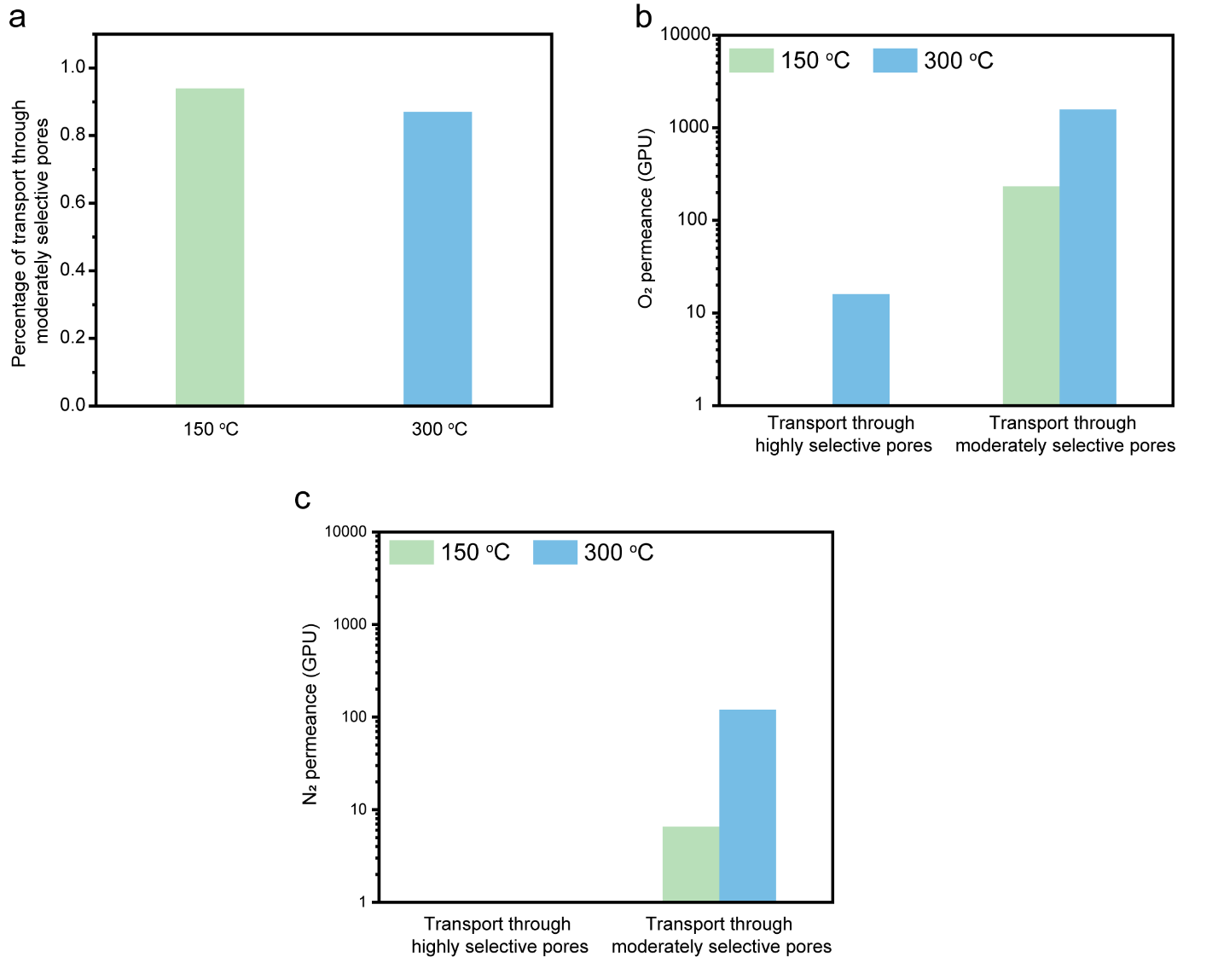


**Figure S16**. (a) Model-calculated fraction of gas transport through moderately selective pores. The model-calculated O_2_ (b) and N_2_ (c) permeance contributes from both highly selective and moderately selective pores. The calculation was based on the graphene membrane after 150 and 300 ^o^C annealing.


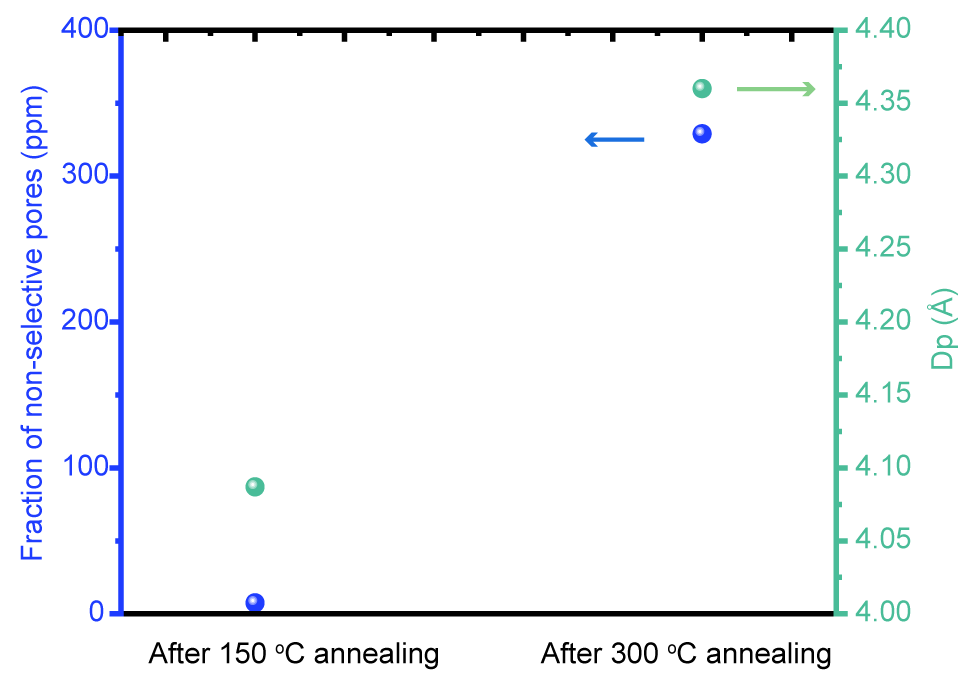


**Figure S17**. The model calculated fraction of non-selective pores and the average effective pore size of graphene after 150 and 300 ^o^C annealing.


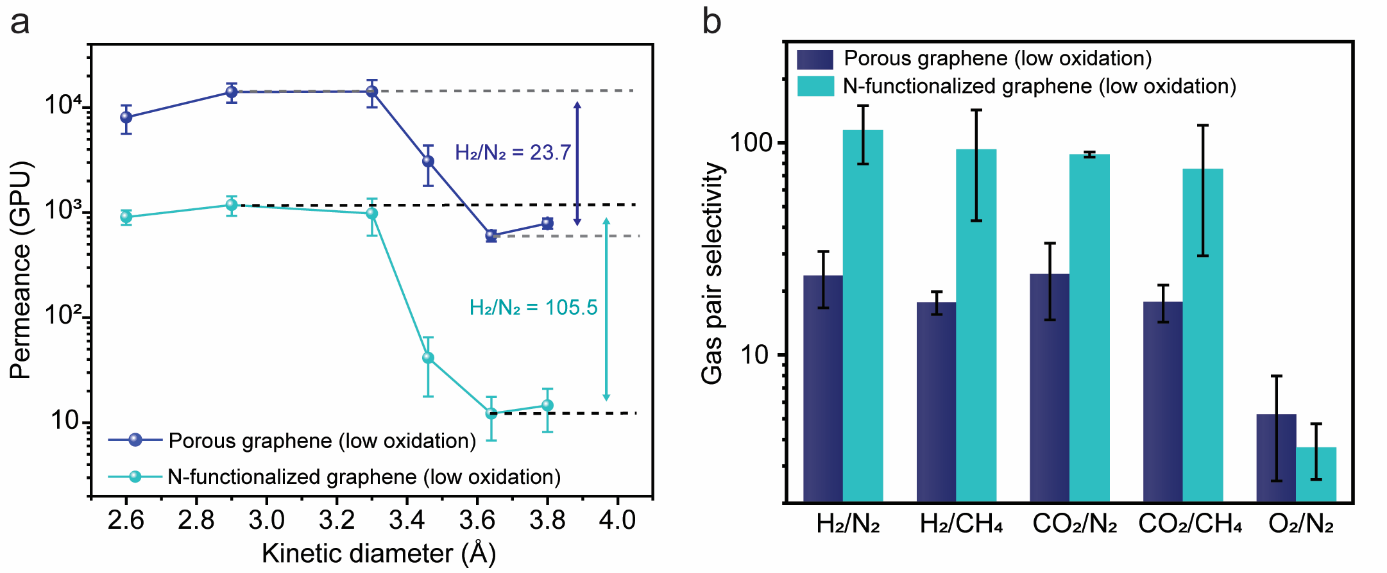


**Figure S18**. Single-gas permeation results of porous graphene and N-functionalized graphene/NPC membranes measured at 30 ºC, 2 bar. The graphene membranes prepared by mild oxidation. The error bars refer to the standard deviation in the separation factor and permeance across three membranes. The center of each error bar represents the average separation factor and permeance calculated from the graphene/NPC membranes.


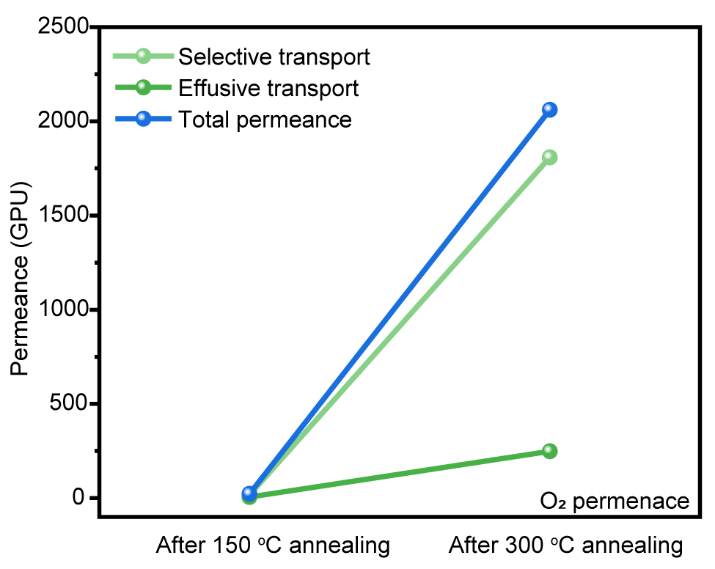


**Figure S19**. Total O_2_ permeance and model-calculated contributions from activated and effusive transport for the same graphene membrane after annealing at 150 ºC and 300 ºC. Gas measurements were carried out at 30 ^o^C at 2 bar.


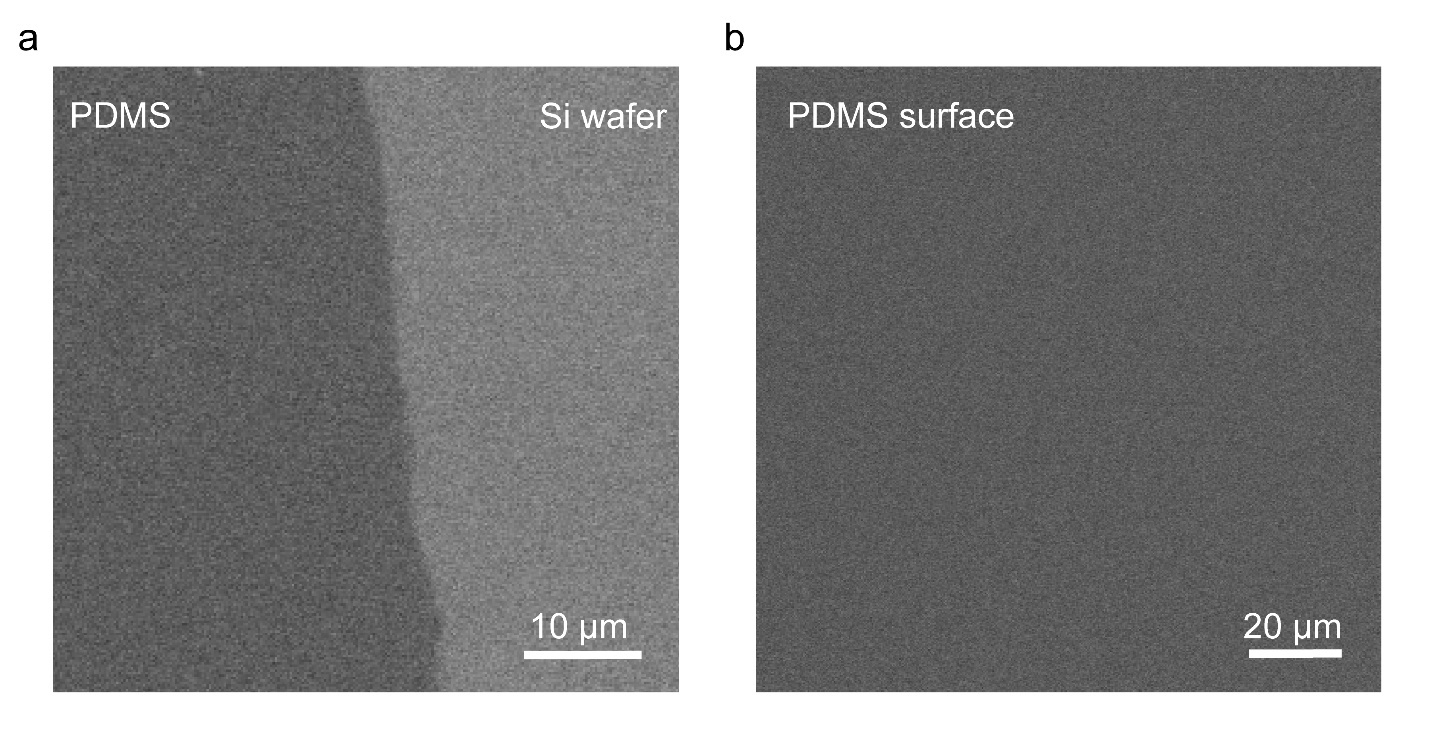


**Figure S20**. SEM characterization of the stand-alone PDMS film surface. SEM images of the surface of PDMS film deposited on silicon wafer in (a) lower magnification and (b) high magnification.


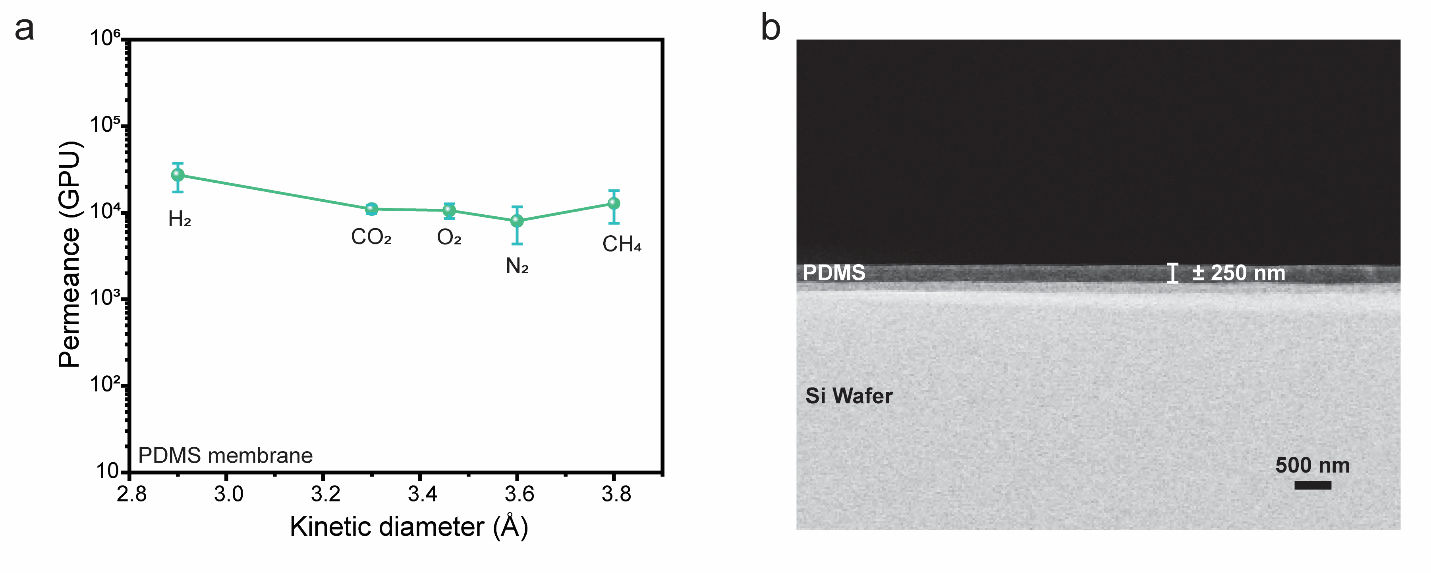


**Figure S21**. (a) Single gas permeation results of PDMS membranes. All the gas measurements were conducted at 30 ^o^C and 2 bar feed pressure. The error bars refer to the standard deviation in the separation factor and permeance across four membranes. The center of each error bar represents the average separation factor and permeance calculated from the PDMS membranes. (b) SEM image of the cross-section of PDMS film deposited on a silicon wafer.


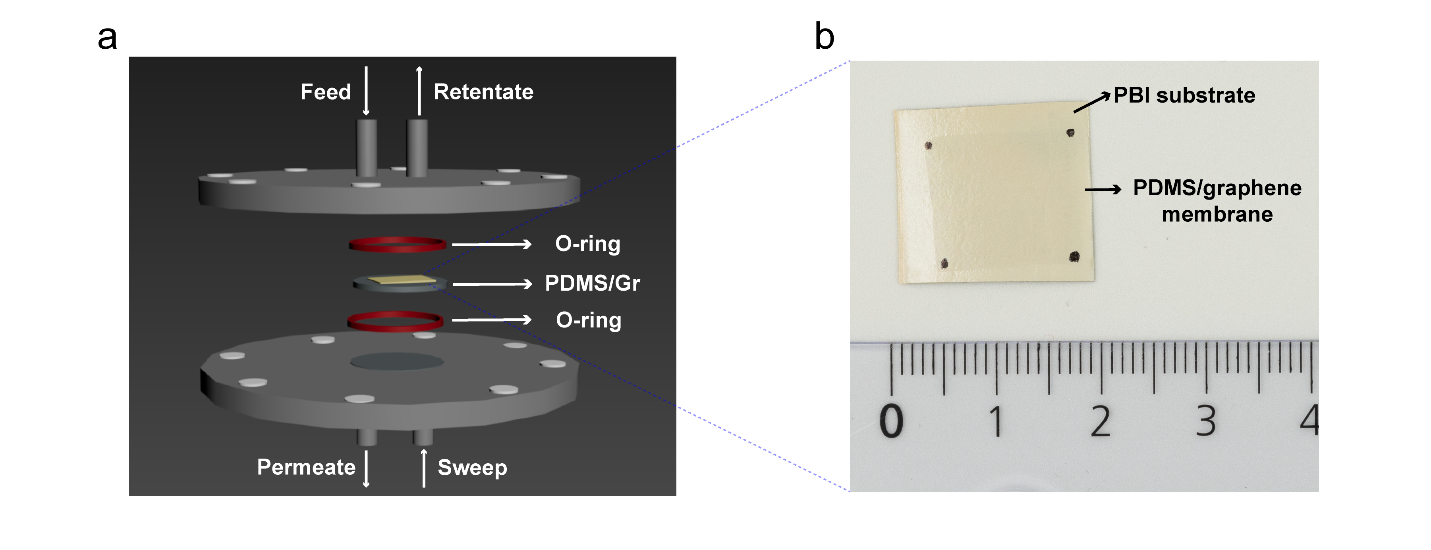


**Figure S22**. Schematic diagram of the membrane module (a) used for the centimeter scale graphene/PDMS membrane (b) test.


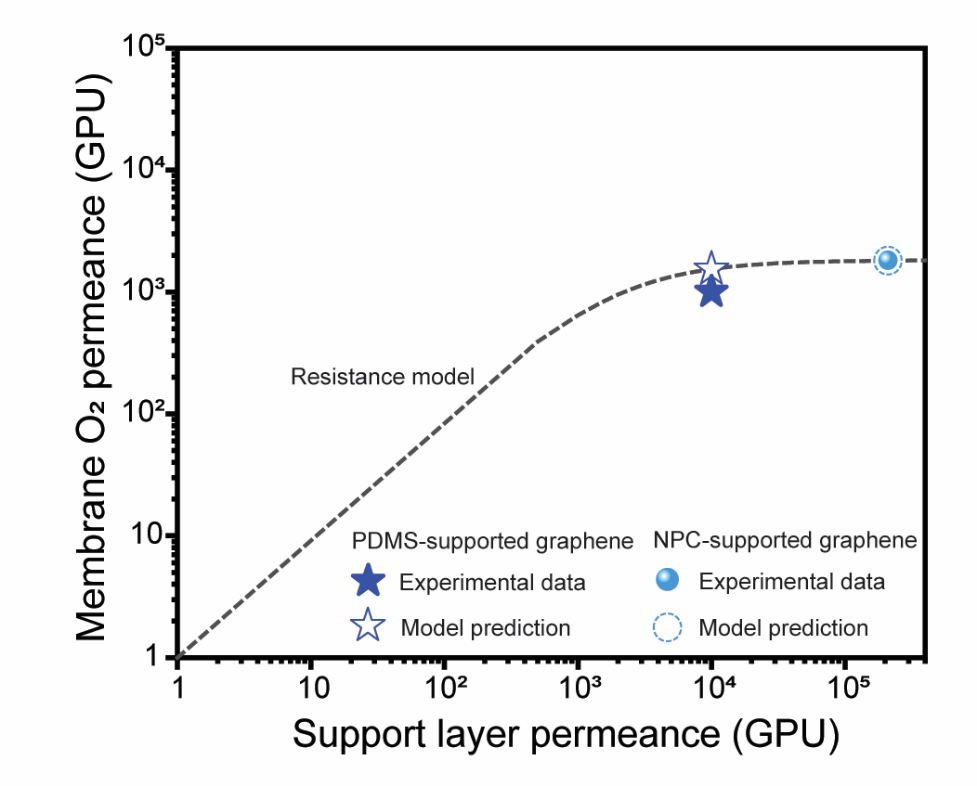


**Figure S23**. Comparison of O_2_ permeance and O_2_/N_2_ separation performance of N-functionalized graphene membrane prepared by two different support films (NPC and PDMS), showing porous graphene controlling gas transport. (a) The evolution of O_2_ permeance of graphene membranes (y-axis) as the function of O_2_ permeance of support film (x-axis). Circles (stars) represent graphene membranes supported by NPC (PDMS). The unfilled star and circle represented the predictions from the gas transport resistance model^[10]^ for graphene membranes. The model (Note S6, Supplementary Information) shows that the transport resistance of the support layer yielding high permeance does not contribute much to graphene membranes.


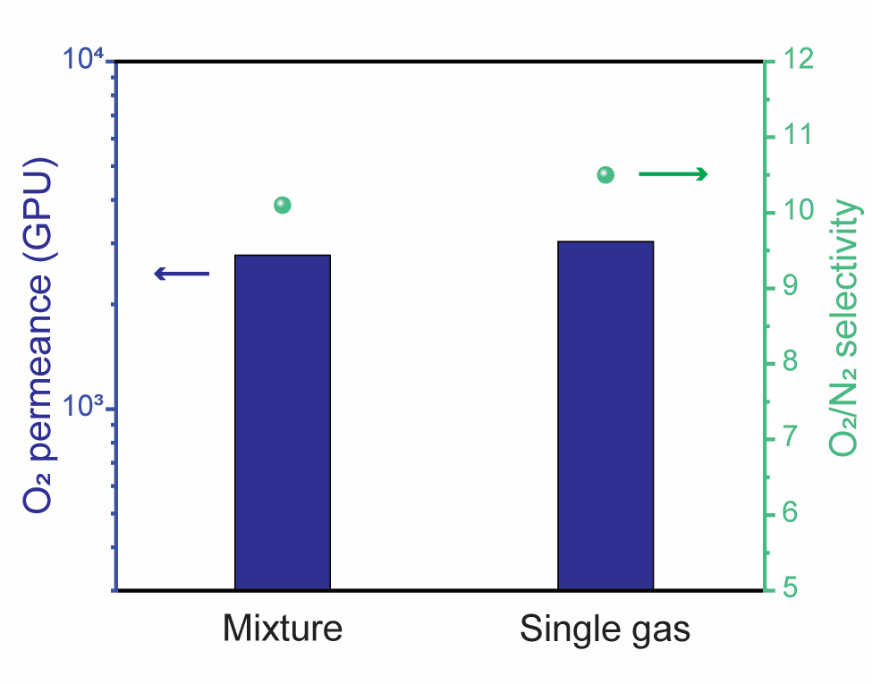


**Figure S24**. Single-gas component and mixture-gas (20% O_2_ in O_2_/N_2_ mixture) permeation results of N-functionalized graphene membranes. All measurements were conducted at 30 ºC and 2 bar feed pressure.


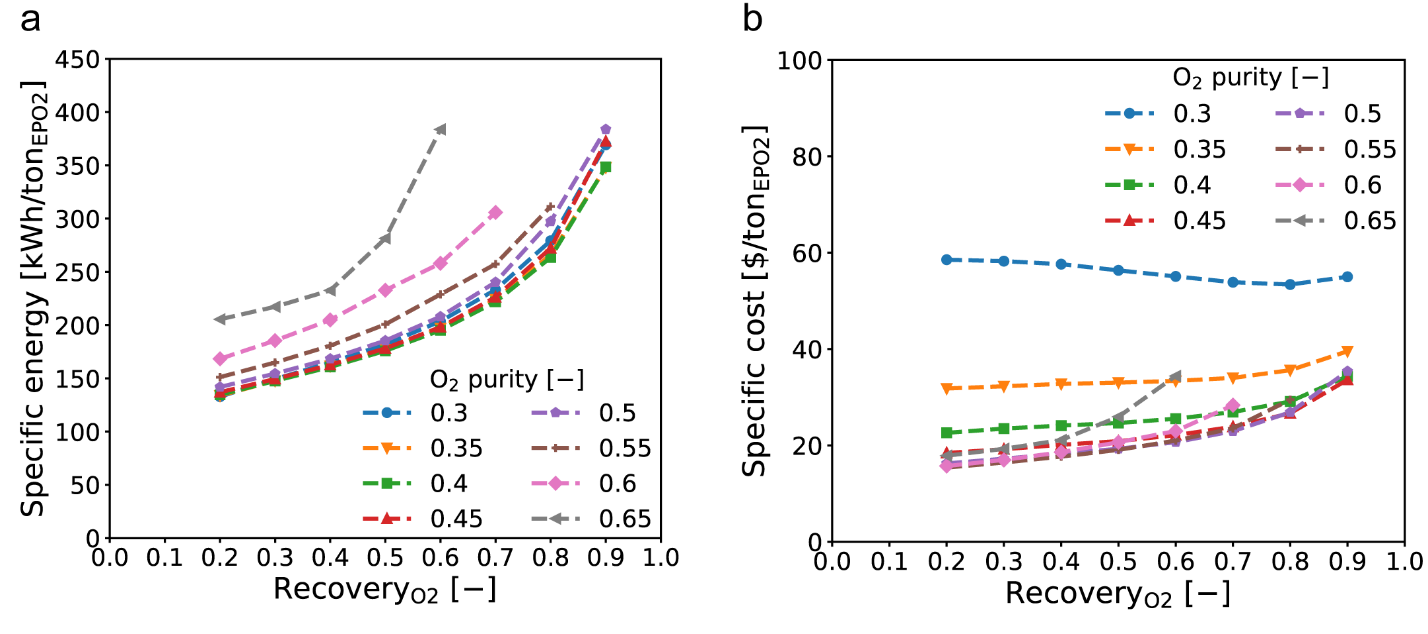


**Figure S25**. The specific energy and cost versus recovery O_2_ concentration under different O_2_ purity conditions.


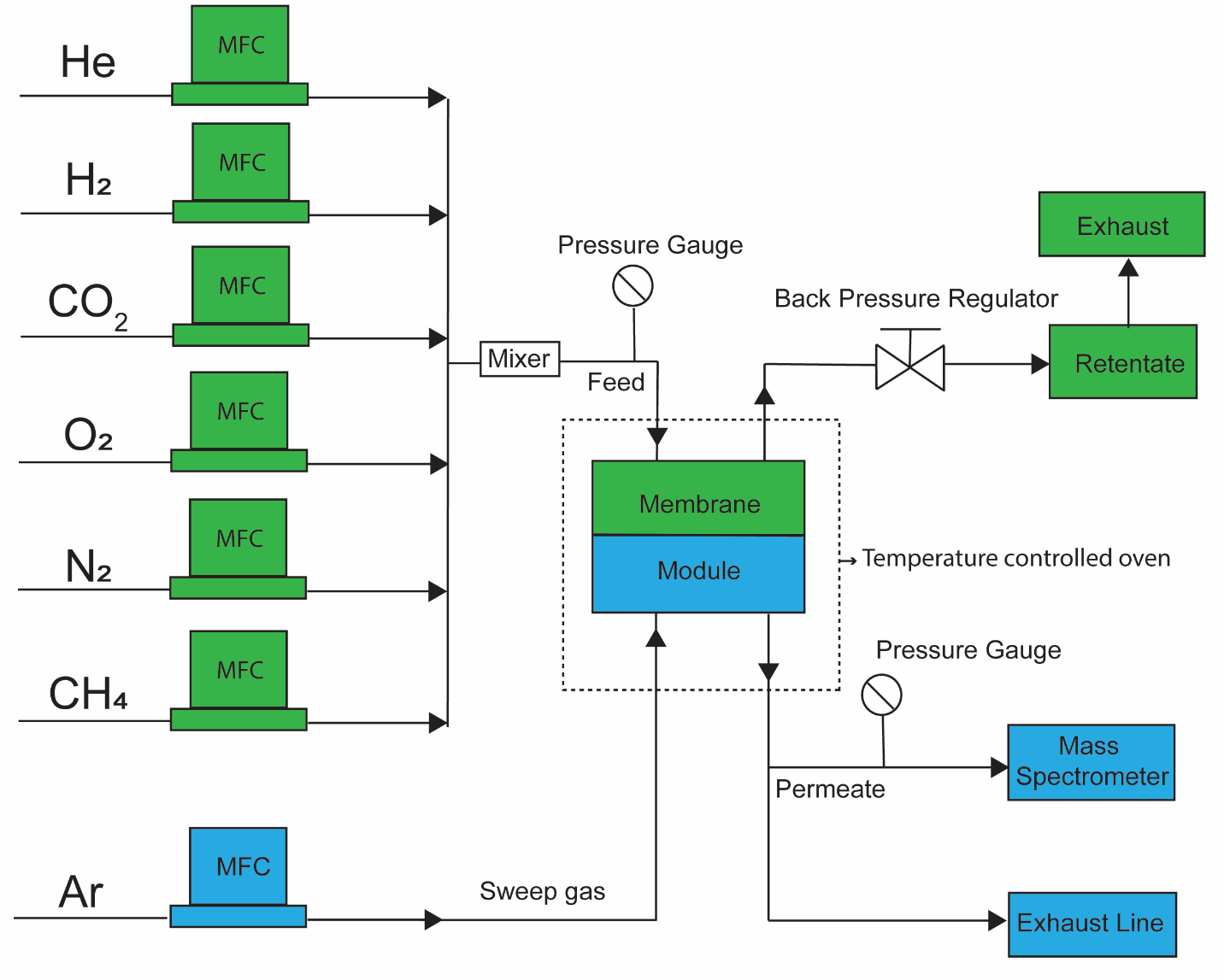


**Figure S26**. Schematic of the gas permeation setup.

**Table S1** The apparent activation energy and activation energy of N-functionalized graphene membrane after 150 and 300 °C annealing.

|  | $E_{app-act}$(kJ mole^-1^) | $E_{act}$ (kJ mole^-1^) |
| --- | --- | --- |
| 150 °C | 2.0 | 27.2 |
| 300 °C | -1.7 | 23.5 |

**Table S2** The MD simulation predicted O_2_ and N_2_ permeability coefficient of highly selective pores and moderately selective pores on the graphene lattice.^[4]^

| Highly selective pores | O_2_ permeability coefficient (mol s^-1^ Pa^-1^) | N_2_ permeability coefficient (mol s^-1^ Pa^-1^) |
| --- | --- | --- |
|  | 3.1×10^-25^ | 0 |
| Moderately selective pores | O_2_ permeability coefficient (mol s^-1^ Pa^-1^) | N_2_ permeability coefficient (mol s^-1^ Pa^-1^) |
|  | 1.7 × 10^-23^ | 1.3 × 10^-24^ |

**Table S3** The model derived highly selective, moderately selective, and total permeable pore density.

| Thermal annealing | Highly selective pore density (cm^-2^) | Moderately selective pore density (cm^-2^) | Total permeable pore density (cm^-2^) |
| --- | --- | --- | --- |
| 150 ^o^C | 1.1×10^11^ | 3.1×10^10^ | 1.4×10^11^ |
| 300 ^o^C | 2.5×10^13^ | 3.1×10^12^ | 2.8×10^13^ |

**Table S4** O_2_/N_2_ mixture separation performance comparison between the N-functionalized graphene membranes presented in this work and state-of-the-art membranes.

| Membrane type | Material | O_2_ permeance  (GPU) | O_2_/N_2_  SF | Reference |
| --- | --- | --- | --- | --- |
| CMS | Cellophane (regenerated cellulose) (550 °C) | 0.145 | 32.3 | ^[12]^ |
|  | Pyrolytic carbon membranes | 75 | 25 | ^[13]^ |
|  | TB-CMS-550 | 41  (4100 barrer) | 5.3 | ^[14]^ |
|  | CMS | 16  (630 barrer) | 10 | ^[15]^ |
|  | 6FDA2:sBPDA0.5:aBPDA0.5/DAM1.8:DABA1.2-  CMS-550 | 40  (2825 barrer) | 6.4 | ^[16]^ |
|  | ODPA-FDA | 34 | 7.0 | ^[17]^ |
| Silica | Silica membranes | 1200 | 3.5 | ^[18]^ |
| Polymer | PIM-C1 | 110 | 3.5 | ^[19]^ |
|  | Tröger’s-base-based polymers | 13 | 5.8 | ^[20]^ |
|  | PDMS-PAN | 860 | 2.2 | ^[21]^ |
|  | PIM-1/Matrimid | 60 | 6.1 | ^[22]^ |
|  | Polysulfone hollow fibers prepared | 8.8 | 5.8 | ^[23]^ |
|  | PIM-TRIP-TB | 8  (1073 barrer) | 5.7 | ^[24]^ |
|  | PIM-EA-TB | 5.4  (971 barrer) | 5.5 | ^[25]^ |
|  | KAUST-PI-1 | 7  (627 barrer) | 5.9 | ^[26]^ |
|  | UV/ozone-modified PIM-1 | 6 | 7 | ^[27]^ |
| Mixed matrix membrane | PIM based MMMs | 13 | 6.3 | ^[28]^ |
|  | Pebax-based MMMs | 3.9 | 12 | ^[29]^ |
|  | MOF-based MMMs | 40 | 8.9 | ^[30]^ |
| Graphene oxide | Graphene oxide | 97  (29 barrer) | 6 | ^[31]^ |
| Single-layer graphene | Oxidized graphene | 1300 | 3.4 | ^[32]^ |
|  | N-functionalized graphene  (150 ^o^C annealed) | 1630 | 6.0 | **This work** |
|  |  | 1930 | 7.1 |  |
|  |  | 2070 | 6.9 |  |
|  |  | 1860 | 5.9 |  |
|  | N-functionalized graphene  (300 ^o^C annealed) | 2770 | 10.0 |  |
|  |  | 3040 | 10.5 |  |
|  |  | 2180 | 10.5 |  |
|  |  | 2330 | 11.6 |  |

**Reference**

[1] L. Wang, M. S. H. Boutilier, P. R. Kidambi, D. Jang, N. G. Hadjiconstantinou, R. Karnik, *Nat. Nanotechnol.* **2017**, *12*, 509.

[2] M. Dakhchoune, X. Duan, L. F. Villalobos, K.-J. Hsu, J. Zhao, M. Micari, K. V. Agrawal, *Ind. Eng. Chem. Res.* **2021**, *60*, 16100.

[3] Z. Yuan, A. Govind Rajan, R. P. Misra, L. W. Drahushuk, K. V. Agrawal, M. S. Strano, D. Blankschtein, *ACS Nano* **2017**, *11*, 7974.

[4] L. Bondaz, A. Ronghe, K. Ganapathy Ayappa, K. V. Agrawal, *Nat. Commun.* **2025**, *16*, 6252.

[5] S. Huang, L. F. Villalobos, S. Li, M. T. Vahdat, H. Chi, K. Hsu, L. S. Bondaz, V. Boureau, N. Marzari, K. V. Agrawal, *Adv. Mater.* **2022**, 2206627.

[6] K.-J. Hsu, L. F. Villalobos, S. Huang, H.-Y. Chi, M. Dakhchoune, W.-C. Lee, G. He, M. Mensi, K. V. Agrawal, *ACS Nano* **2021**, *15*, 13230.

[7] K.-J. Hsu, H.-Y. Chi, Y. Shen, S. Huang, R. Goswami, K. V. Agrawal, *Adv. Funct. Mater.* **2025**, 2503121.

[8] M. Micari, K. V. Agrawal, *J. Memb. Sci.* **2022**, *641*, 119883.

[9] H. Lin, M. Zhou, J. Ly, J. Vu, J. G. Wijmans, T. C. Merkel, J. Jin, A. Haldeman, E. H. Wagener, D. Rue, *Ind. Eng. Chem. Res.* **2013**, *52*, 10820.

[10] H. B. Park, J. Kamcev, L. M. Robeson, M. Elimelech, B. D. Freeman, *Science* **2017**, *356*, eaab0530.

[11] K.-J. Hsu, S. Li, M. Micari, H.-Y. Chi, L. F. Villalobos, S. Huang, L. Zhong, S. Song, X. Duan, A. Züttel, K. V. Agrawal, *Nat. Energy* **2024**, DOI 10.1038/s41560-024-01556-0.

[12] S. C. Rodrigues, M. Andrade, J. Moffat, F. D. Magalhães, A. Mendes, *J. Memb. Sci.* **2019**, *572*, 390.

[13] H. B. Park, S. Y. Lee, Y. M. Lee, *J. Mol. Struct.* **2005**, *739*, 179.

[14] Z. Wang, H. Ren, S. Zhang, F. Zhang, J. Jin, *ChemSusChem* **2018**, *11*, 916.

[15] Y. K. Kim, H. B. Park, Y. M. Lee, *J. Memb. Sci.* **2004**, *243*, 9.

[16] Z. Liu, W. Qiu, W. Quan, W. J. Koros, *Nat. Mater.* **2023**, *22*, 109.

[17] C.-P. Hu, C. K. Polintan, L. L. Tayo, S.-C. Chou, H.-A. Tsai, W.-S. Hung, C.-C. Hu, K.-R. Lee, J.-Y. Lai, *Carbon* **2019**, *143*, 343.

[18] R. Izumi, N. Moriyama, K. Ishizaki, H. Nagasawa, T. Tsuru, M. Kanezashi, *J. Memb. Sci.* **2024**, *691*, 122228.

[19] J. Zhang, H. Kang, J. Martin, S. Zhang, S. Thomas, T. C. Merkel, J. Jin, *Chem. Commun.* **2016**, *52*, 6553.

[20] Z. Zhu, J. Zhu, J. Li, X. Ma, *Macromolecules* **2020**, *53*, 1573.

[21] P. Li, H. Z. Chen, T.-S. Chung, *J. Memb. Sci.* **2013**, *434*, 18.

[22] W. F. Yong, F. Y. Li, Y. C. Xiao, T. S. Chung, Y. W. Tong, *J. Memb. Sci.* **2013**, *443*, 156.

[23] S. C. Pesek, W. J. Koros, *J. Memb. Sci.* **1994**, *88*, 1.

[24] M. Carta, M. Croad, R. Malpass-Evans, J. C. Jansen, P. Bernardo, G. Clarizia, K. Friess, M. Lanč, N. B. McKeown, *Adv. Mater.* **2014**, *26*, 3526.

[25] M. Carta, R. Malpass-Evans, M. Croad, Y. Rogan, J. C. Jansen, P. Bernardo, F. Bazzarelli, N. B. McKeown, *Science* **2013**, *339*, 303.

[26] B. S. Ghanem, R. Swaidan, E. Litwiller, I. Pinnau, *Adv. Mater.* **2014**, *26*, 3688.

[27] Q. Song, S. Cao, P. Zavala-Rivera, L. Ping Lu, W. Li, Y. Ji, S. A. Al-Muhtaseb, A. K. Cheetham, E. Sivaniah, *Nat. Commun.* **2013**, *4*, 1918.

[28] L. Hao, K.-S. Liao, T.-S. Chung, *J. Mater. Chem. A* **2015**, *3*, 17273.

[29] X. Cao, R. Song, L. Zhang, F. Cheng, Z. Wang, *J. Memb. Sci.* **2024**, *698*, 122624.

[30] Z. Qin, Y. Sun, Z. Zhang, C. Zhang, C. Tang, C. Geng, Z. Qiao, *Chem. Eng. J.* **2024**, *497*, 154615.

[31] H. W. Kim, H. W. Yoon, S.-M. Yoon, B. M. Yoo, B. K. Ahn, Y. H. Cho, H. J. Shin, H. Yang, U. Paik, S. Kwon, J.-Y. Choi, H. B. Park, *Science* **2013**, *342*, 91.

[32] S. Huang, S. Li, L. F. Villalobos, M. Dakhchoune, M. Micari, D. J. Babu, M. T. Vahdat, M. Mensi, E. Oveisi, K. V. Agrawal, *Sci. Adv.* **2021**, *7*, eabf0116.
